# Delivery of a novel membrane-anchored Fc chimera enhances NK cell-mediated killing of tumor cells and persistently virus-infected cells

**DOI:** 10.1371/journal.pone.0285532

**Published:** 2023-05-05

**Authors:** Namita Varudkar, Elisabeth M. Shiffer, Jeremiah L. Oyer, Alicja Copik, Griffith D. Parks

**Affiliations:** Burnett School of Biomedical Sciences, College of Medicine, University of Central Florida, Orlando, FL, United States of America; University of Northern Colorado, UNITED STATES

## Abstract

Antibody-dependent cellular cytotoxicity (ADCC) is one of the most powerful mechanisms for Natural Killer (NK) cells to kill cancer cells or virus-infected cells. A novel chimeric protein (NA-Fc) was created, which when expressed in cells, positions an IgG Fc domain on the plasma membrane, mimicking the orientation of IgG bound to the cell surface. This NA-Fc chimera was tested with PM21-NK cells, produced through a previously developed particle-based method which yields superior NK cells for immunotherapeutic applications. Real time viability assays revealed higher PM21-NK killing of both ovarian and lung cancer cells expressing NA-Fc, which correlated with increased release of TNF-α and IFN-γ cytokines from NK cells and was dependent on CD16-Fc interactions. Lentivirus delivery of NA-Fc to target cells increased the rate of PM21-NK cell killing of A549 and H1299 lung, SKOV3 ovarian and A375 melanoma cancer cells. This NA-Fc-directed killing was extended to virus infected cells, where delivery of NA-Fc to lung cells that were persistently infected with Parainfluenza virus resulted in increased killing by PM21-NK cells. In contrast to its effect on PM21-NK cells, the NA-Fc molecule did not enhance complement mediated lysis of lung cancer cells. Our study lays the foundation for application of the novel NA-Fc chimera that could be delivered specifically to tumors during oncolytic virotherapy to mark target cells for ADCC by co-treatment with adoptive NK cells. This strategy would potentially eliminate the need to search for unique cancer specific antigens for development of new antibody therapeutics.

## Introduction

Antibodies (Abs) are an important part of immune responses which can recognize tumor cells or virus infected cells to mediate clearance [1,2]. There is intense interest in the development of new antibody-based therapies, including monoclonal (mAb), bi-specific and tri-specific Abs [3]. A large number of therapeutic mAbs have been developed and are being used to treat various types of cancers [4–6]. Likewise, Ab therapies against viral infections have seen a rapid increase in applications, including their use against acute infections such as SARS-CoV-2 [7] or persistent infections such as HIV [8].

Ab-based therapies may function through more than one mechanism of action, such as induction of immune cell functions, activation of complement, blocking of cell growth, marking of cancer cells to facilitate immune cell recognition and killing, and blocking immune checkpoint inhibitors [4,9]. One of the most important functions of therapeutic Abs is their potential ability to trigger killing of tumor cells or virus infected cells, through direct binding of IgG or other Ab types to the target cell surface to activate immune cell functions. The structure of IgG includes a COOH-terminal region known as the fragment crytallizable (Fc) domain [4,10], and this region is responsible for driving Ab-dependent cellular cytotoxicity (ADCC).

Among the important cell types that carry out ADCC, Natural Killer (NK) cells play pivotal roles in immune-mediated clearance of cells [11–13], acting through cell surface receptors which recognize signatures on target cancer cells or virus infected cells. When Abs are opsonized on the surface of tumor cells or virus infected cells, their C-terminal Fc region is recognized by the NK cell through the FcγIIIA receptor (CD16) [6]. This CD16-Fc interaction triggers functional and phenotypic changes in NK cells, including NK cell activation and targeted release of cytotoxic granules containing perforin and granzyme during the process of degranulation [14,15] and lysis of target cells along with secretion of cytokines such as IFN-γ and TNF-α [16,17]. Yet, many cancer patients experience NK cell deficiency either due to the cancer itself or follow up treatments, which can hinder the efficacy of antibody-based therapies [18]. Furthermore, some therapeutic antibody can target antigens that are also expressed on NK cells *e*.*g*. CD38 which can lead to NK cell fratricide and their depletion [19].

NK cell adoptive therapy is a promising approach to cancer and infectious disease immunotherapy, which can be combined with therapeutic antibodies to enhance their efficacy [13]. NK cells are part of innate immunity and their function is regulated by the balance of activating and inhibitory signals received from a plethora of surface receptors allowing them to distinguish self from foreign as well as healthy from diseased or cancerous cells [18]. Due to these innate mechanisms, NK cells are an attractive cell population with broad anti-tumor activity and great safety profiles. Adoptively transferred NK cells have been found be safe even if derived from allogeneic or haploidentical mismatched donors allowing for development of safe off-the-shelve sources of NK cells for potential therapeutic use [20–24].

There are currently large number of clinical trials testing the therapeutic potential of utilizing adoptive allogeneic NK cells for treatment of cancers as well viral diseases. This rapid expansion of clinical trials of NK cells was facilitated by advancing methods of NK cell expansion and *in vitro* culture. We have developed a particle-based method for *ex vivo* expansion of human NK cells that yields highly cytotoxic NK cells [25,26]. This involves generation of particles (PM21-) derived from an engineered K562 cell line that expresses the NK cell stimulating ligands 41-BBL and membrane bound IL-21 [25,26]. These PM21 particles stimulate specific *in vitro* expansion of NK cells from unselected PBMCs, resulting in >1,000-fold NK cell expansion and with >90% purity after 2 weeks of culture [25,26]. This platform produces vastly superior NK cells, with ~10-100-fold higher cytotoxicity than NK cells generated by activation with high concentration of IL2. We have shown that these NK cells are effective in reducing ovarian tumor load in mice models [27]. PM21-NK cells are currently being tested in a clinical trial for treatment of leukemia (NCT04395092).

Although Abs can be effective in terms of cancer and infectious disease treatments, there can also be limitations to their use, including resistance and relapse of tumors, off target effects, and the cost and time to develop a mAb therapy. Further challenges can be in their inherent properties of their large size which may contribute to inadequate pharmacokinetics, tissue accessibility, and impaired diffusion [28]. It is likely that one of the greatest challenges will be in defining specific tumor or viral antigens to target with mAbs but not normal tissues, with many tumors lacking clearly unique targetable antigens suitable for therapeutic development [5].

In this study, we propose a novel approach to target an antibody mimic to the surface of cancer cells to mark them for recognition and killing by adoptively transferred NK cells. This approach would allow to potentially treat any type of cancer that can be specifically targeted by delivery mechanisms such as oncolytic viruses (OVs) and would get around the need of unique cancer antigens. While a number of immune cell types can recognize antibodies bound to target cell surfaces, the goal of this current study was to provide proof of concept that NK cell activity could be enhanced *in vitro* through Fc-driven mechanisms in the absence of an antigen-specific Ab. We hypothesized that expression of a membrane-bound Fc-containing protein on the surface of target cancer cells could mark them for NK cell-mediated cell killing.

We have used our prior knowledge of the topology of type II integral membrane proteins [29], the minimal signals required for transport of some membrane proteins to the cell surface [30] and the structure of the influenza virus Neuraminidase (NA) protein [31] to create a novel chimeric NA-Fc protein which positions the IgG Fc domain on the plasma membrane with an N_in_-C_out_ orientation–mimicking the orientation of IgG bound to the cell surface. Using in vitro real-time assays for cell viability, we show that cancer cells stably expressing this NA-Fc protein have an increased rate and extent of PM21-NK cell killing through CD16-Fc dependent mechanisms. Most importantly, delivery of the NA-Fc chimera to a range of different types of tumor cells or to virus persistently infected cells increased their killing by PM21-NK cells in vitro. Our results provide proof of principle of a novel mechanism to target cancer or virus-infected cells for enhanced NK cell killing.

## Materials and methods

### Cells and viruses

Cultures of A549 (ATCC) and CV-1 cells were grown in Dulbecco modified Eagle medium (DMEM) supplemented with 10% heat inactivated fetal bovine serum (HI FBS, HI FBS, Gibco, Thermo Fisher Scientific, Waltham, MA, USA) at 37°C under humidified, 5% CO2 atmosphere. Nuc Light Red A549 cells expressing a nuclear red fluorescent protein (NLR-A549 cells) were purchased from Sartorius (Sartorius, Göttingen, Germany). H1299-NLR, SKOV3-NLR and A375-NLR cells were generated by transduction with NLR- lentivirus (Sartorius) and cultured in RPMI with 10% FBS and 1 μg/ml puromycin. The P/V mutant (P/V virus) virus expressing green fluorescence protein (GFP) has been described [32]. Virus titers were determined by plaque assay on CV-1 cells.

### Generation of cell lines stably expressing NA-Fc

**Fig 1:**
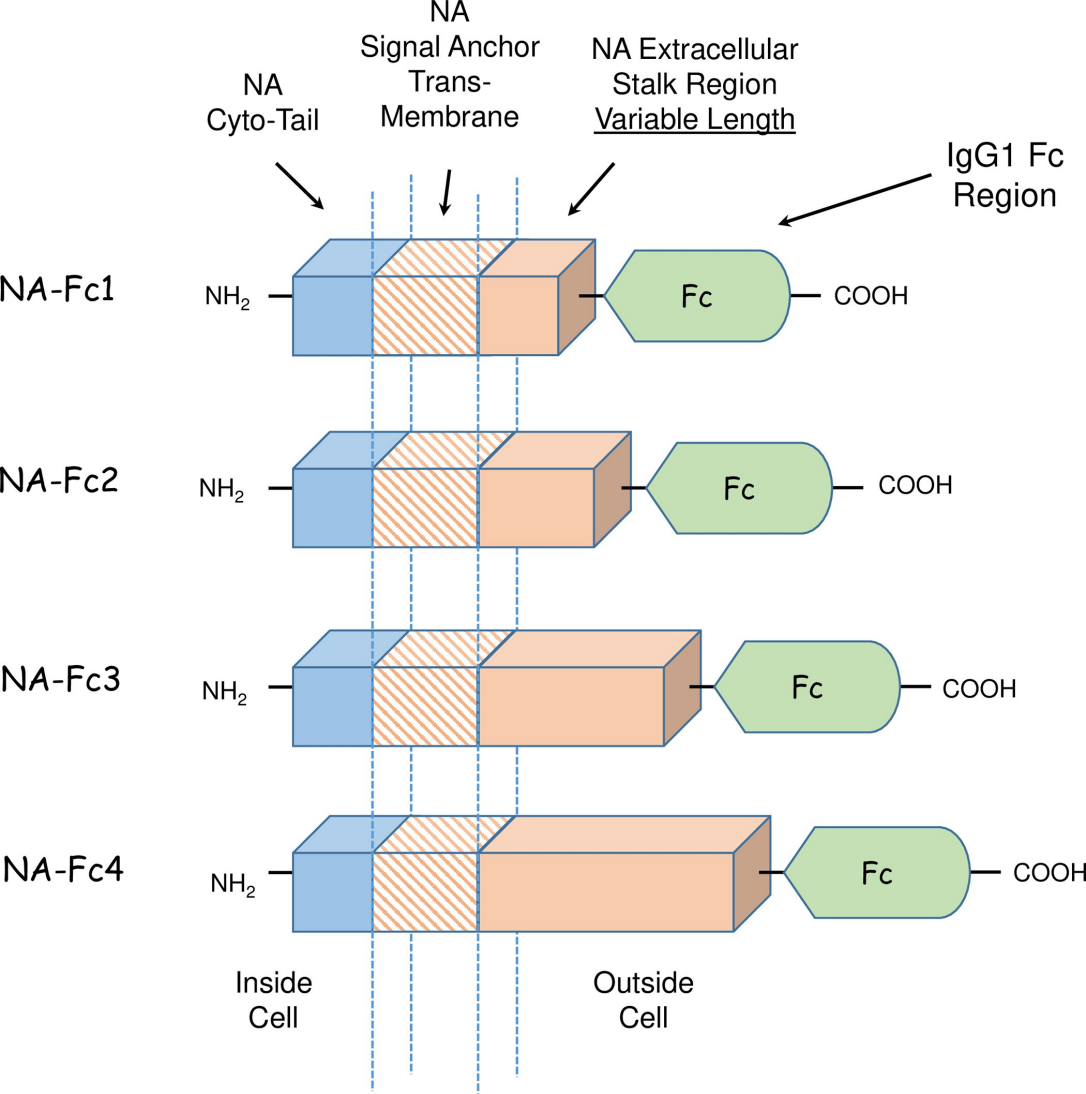
Schematic structures and membrane orientation of NA-Fc chimeras. A series of 4 plasmids were constructed to encode chimeric proteins consisting of the NA cytoplasmic tail, transmembrane signal-anchor and stalk region fused to the IgG1 Fc region. NA-Fc1 through NA-Fc4 differ only in the length of the extracellular stalk region and increases the distance of the Fc domain from the plasma membrane.

To construct plasmids expressing the NA-Fc chimera, Vero cells were infected with PR8 Influenza A virus and total RNA was purified using TRIzol (Invitrogen, Thermo Fisher Scientific, Waltham, MA, USA). RNA (1μg) was used to generate cDNA by reverse transcription- PCR. Primers for amplification were designed with restriction enzymes ECORI and BGlII to sites to generate 4 different constructs (Fig 1) which differ only in the length of the NA stalk region as described in the results section below. PCR products were cloned into the ECORI and BGlII sites of pFUSE-hIgG1-Fc1 (Invivogen, San Diego, CA, USA) which encodes a multiple cloning site upstream of DNA encoding an Fc fragment. SKOV3 cells were transfected with each of these plasmids (NA-Fc1, NA-Fc2, NA-Fc3 & NA-Fc4) followed by bulk sorting of cells using APC conjugated anti-human IgG Fc (clone M1310G05) antibody (Biolegend, San Diego, CA, USA).

As an alternative approach, the NLR cell lines stably expressing NA-Fc proteins 1–4 were generated by transducing the SKOV3-NLR and A549-NLR with NA-Fc4 Lentivirus (LV). In brief, the NA-Fc4 LV was made by transfecting 293T cells with NA-Fc4 plasmid (Vector Laboratories, Burlingame, CA, USA) along with plasmids encoding VSV-G envelope (pMD2.G) and 2nd generation lentiviral packaging (ps PAX2) plasmids. The supernatant containing LV was collected at 24 and 48 hours (h) post transfection and combined.

### PM21-NK cell preparation

PM21-particles were generated, and NK cells were expanded from peripheral blood mononuclear cells (PBMCs) as described previously [32]. Briefly, PBMCs were depleted of T-cells (EasySep CD3 selection kit; STEMCELL Technologies Inc., Vancouver CA) and then cultured for up to 25 days (d) with 100 U/mL Interleukin-2 (IL-2; Peprotech Inc., Cranbury, New Jersey, USA) and 200 μg/mL of PM21-particles in SCGM media (CellGenix, Freiburg im Breisgau, Germany) supplemented with 10% non-HI FBS. PM21 particles were derived from CSTX-002 (K562-nmIL21-41BBL) cells, provided by Kiadis Pharma, a Sanofi company and maintained in RPMI media supplemented with 10% FBS. PM21-NK cells were tested with target cells at different Effector:Target (E:T) cell ratios, to assure optimal cytotoxicity for different experimental conditions described in the text and to account for variations between donor NK cells.

### Cytotoxicity and cell killing assays

Flow cytometric cytotoxicity assays were carried out as previously described [27]. Briefly, target cells were plated in RPMI containing 10% non-HI FBS (NK cell media) at 30,000 cells per 50 μL in polystyrene U-bottom plates. NK cells were added at different E:T cell ratios in 50 μL and were co-incubated with target cells for 30–45 min in NK cell media containing IL-2. Cells were then washed with PBS- and stained with Annexin V (Biolegend). Cytotoxicity was calculated based on the total number of Viable Target Cells (TFL4^+^/Annexin V^-^) remaining in each well with effectors (VTC ^E:T^) and referenced to average VTC in “target alone” control wells (VTC ^T ctrl^ Cytotoxicity ^E:T^ (%) = (1-VTC ^E:T^ / AverageVTC ^Tctrl^) X 100 (32).

For the Incucyte S3 Live-Cell Analysis system (Sartorius), naïve NLR and NA-Fc4NLR-expressing target cells were plated in triplicate in 96-well plates (Corning, NY, USA) at 7,000 cells/well, and left in the incubator overnight. Cells were washed with PBS before addition of NK cells at various E:T ratios in NK cell media. Plates were maintained in the Incucyte system at 37°C for 3–4 d, while images were captured every 2 h using 10x objective in red and phase channels. Target cell growth/killing was monitored over time and was normalized to initial number of cells present at the time of NK cell addition. For this, the number of viable cells remaining in the well was quantified for each time point based on Red Object Count (ROC) normalized to the value at time 0 (ROC/ROC _t(0h)_) when NK cells were initially added to co-cultures (32). The length of time of recording loss of ROC shown in each figure varies, depending on the time required for different experimental conditions to result in a plateauing of cytotoxicity.

For transduction experiments, 200,000 naïve NLR target cells were transduced with either BFP (blue fluorescent protein) LV or NA-Fc4 LV in the presence of 10 μg/ml of polybrene (Millipore Sigma, Burlington, MA, USA). Two days after transduction the cells were trypsinized, their transgene efficiency was determined and cytotoxicity assay was performed on IncuCyte as described above.

### Degranulation assay and cytokine quantification

For degranulation assays, expanded PM21-NK cells were incubated with either targets at 1:1 E:T ratio (50,000 cells each) or treated with PMA/Ionomycin drugs (for positive control) for 16 h at 37°C, 5% CO2 in the cell incubator in flat bottom 96 well plates. PE-conjugated anti-human CD107a antibody (Biolegend) was added to each of the reactions at the beginning of the assay and Golgi-monensin (Biolegend) was added at a concentration of 0.67 μl/ml after 1 hour (h) of incubation. After incubation, cells were stained with a cocktail of antibodies containing anti-human CD56-Pacific blue clone (MEM-188), CD3-FITC clone (OKT3) and CD107a-PE clone (H4A3) all from Biolegend. The cells were washed once with PBS- and analyzed on flow cytometer. The gating strategy for measuring CD56 is shown in S1 Fig. where there a homogeneous population of CD56+ cells was seen.

For cytokine analysis, NK cells were co- incubated with vehicle, drugs or target cells as described above. As a positive control, they were stimulated with cytokine cocktail (10 ng/mL IL12, 50 ng/mL IL18, 100 ng/mL IL15). All reactions were done in triplicate in 96-well flat bottom plates in the presence of Brefeldin A (Biolegend) added at a concentration of 1μl/ml. Six h post incubation, the cells were fixed, and permeabilized using eBioscience Intracellular Fixation and Permeabilization Buffer, according to the manufacturer’s instructions (Invitrogen, Thermofisher Scientific). Cells were stained for 15 mins with an antibody cocktail containing anti-human CD56-PE, CD3-FITC, IFNγ-Pacific blue and TNFα-APC. The cells were washed once with PBS- and analyzed on flow cytometer CytoFLEX (Beckman Coulter, Brea, CA, USA) using CytExpert software (Beckman Coulter).

### Receptor blocking and complement assays

Target naïve and NA-Fc4 A549-NLR cells were plated as described previously and were incubated for 1 h with 10 μg/ml unconjugated anti-human IgG Fc antibody. After co-culturing with PM21-NK cells, cytotoxicity assays were performed on Incucyte instrument as mentioned above [32].

For experiments blocking NK cell receptor, NK cells were incubated with 10 μg/ml unconjugated antibodies to NK cell CD16 receptor (Biolegend) for 1 h at 37°C before incubation with naive and NA-Fc4 A549-NLR cells. The cytotoxicity assays were performed on Incucyte instrument as described above [32].

For complement experiments, both target naïve and NA-Fc4 expressing A549-NLR cells were treated with 20% normal human serum (NHS, Cat no. 28425-Innovative Research, Novi, MI, USA), 20% heat inactivated NHS (HI NHS) as a control for complement activity or left untreated. As a positive control, both sets of target cells were infected with parainfluenza virus 5 (PIV5) mutant at an MOI of 10 for 16 hours and then treated with 20% NHS or left untreated. The extent of cytotoxicity was determined on IncuCyte instrument as described in the NK cell cytotox assay. Images were captured every 2 h and ROC was recorded and normalized to the t0 ROC before addition of complement.

### Statistical analyses

Statistical analysis was carried out with the assistance of faculty within the UCF College of Medicine Biostatistics Group. Statistical analysis was performed using two-way ANOVA test and by applying the Tukey’s or Dunett’s post hoc test in time courses using GraphPad Prism software as detailed in Fig legends. No outliers were removed from the analysis of data. All Incucyte experiments were performed in triplicate and with experimental repeats. No assumptions in statistical models of time courses as a whole or power analyses were performed, but statistical analysis as described above was done comparing one timepoint between individual experimental conditions. In figures from Incucyte timecourses, stars indicate the point where the *p*-values first begin, and this statistical significance extends for later timepoints until a new star appears with a different p value.

## Results

### PM21-NK cells effectively kill ovarian tumor cells expressing NA-Fc chimeras

In this study, an N-terminally membrane-anchored Fc chimeric protein was designed that mimicked the orientation of surface-bound IgG antibody (Fig 1). The rational was that the influenza A virus Neuraminidase (NA) protein could serve to orient the Fc domain at the cell surface, given that NA protein has an N-terminal cytoplasmic domain, internal un-cleaved signal-anchor and C-terminal extracellular domain (31)–mimicking the C-terminal “outside” orientation of surface bound IgG. Thus, cDNA plasmids were constructed encoding the NA cytoplasmic tail, transmembrane signal-anchor and extracellular stalk region fused to the C-terminal 223 amino acids from the Fc domain of IgG1 (See Fig 1 above). With *a priori* knowledge on requirements for transport, stability and NK cell recognition of this hybrid protein, a series of NA-Fc chimeras were constructed to have varying lengths of the external NA stalk region (Fig 1), with 15, 45, 65 and 85 residues for NA-Fc1 through–Fc4, respectively.

**Fig 2:**
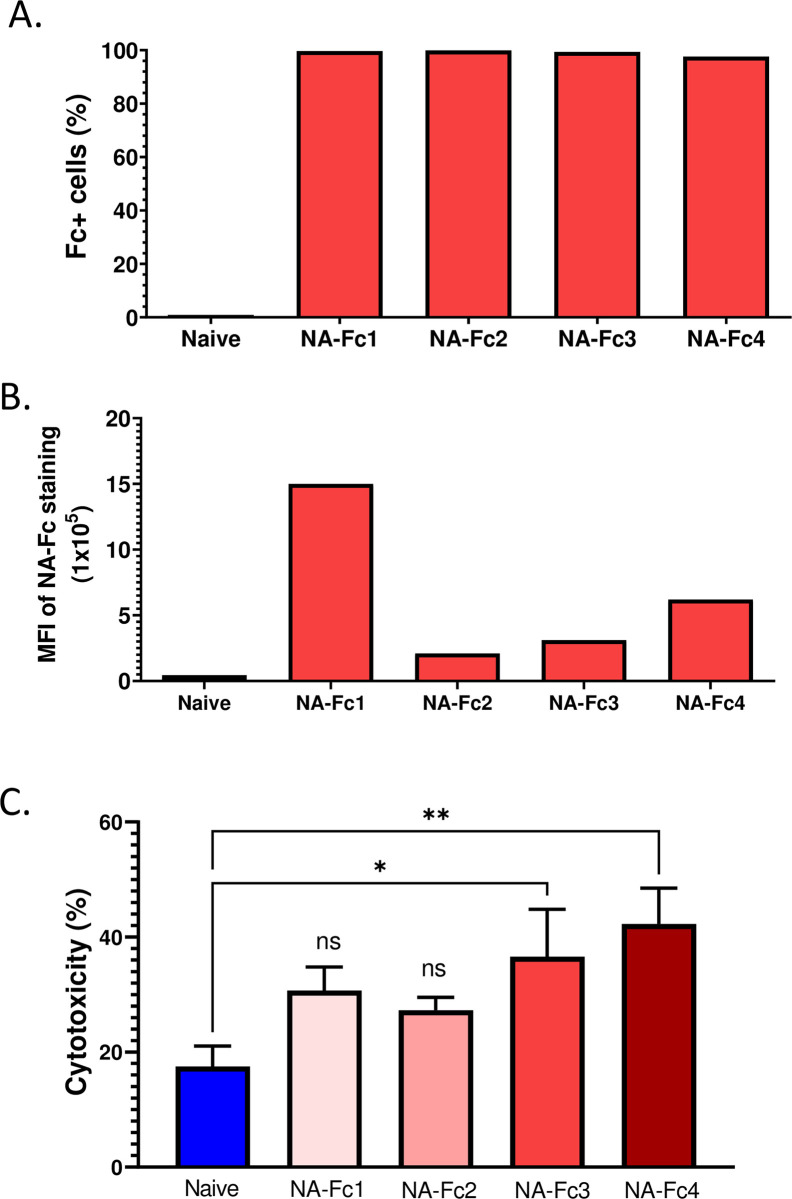
PM21-NK cells effectively lyse ovarian cancer cells expressing the NA-Fc chimeras. (A, B) SKOV3 ovarian cells were transfected with cDNAs encoding one of the four NA-Fc chimeras. Cell lines were stained with antibody to IgG1 Fc and sorted by flow cytometry. Naïve and transfected SKOV4cells were stained with antibody to Fc domain and percent Fc+ (panel A) and MFI (panel B) for surface expression was assayed by flow cytometry. (Panel C) PM21-NK cells were incubated with cultures of naïve SKOV3 cells or one of the four cell lines expressing NA-Fc for 45 mins at an E:T of 1:1. Percent cytotoxicity was determined using a flow based cytometric assay as described in Material & Methods. Data is representative of 4 independent experiments conducted with 2 different NK cell donors with triplicates and error bars represent SD. Data was analyzed using two-way ANOVA analysis. * and ** indicate p values of p<0.05 and p<0.01.

To determine cell surface expression of the chimeras, human ovarian SKOV3 tumor cells were transfected with a plasmid expressing one of the four NA-Fc chimeras, bulk-sorted by FACS (fluorescent activated cell sorting) and stained for levels of surface IgG1 Fc. As shown in Fig 2A, ~95–98% of cells expressed surface Fc across the four cell lines. Importantly however, a comparison of mean fluorescent intensity (MFI) between cell lines (Fig 2B) showed that surface Fc expression levels differed, with a gradient of expression levels from NA-Fc1, NA-Fc4, NA-Fc3 down to the lowest levels with NA-Fc2.

To test if these Fc expressing SKOV3 tumor cells are killed by PM21-NK cells, naive SKOV3 cells or the four NA-Fc-expressing stable SKOV3 cell lines were co-cultured with PM21- NK cells at a ratio of 1:1 and cytotoxicity of the target SKOV3 cells was determined using a flow-based assay (see materials and methods). As shown in Fig 2C, naïve SKOV3 tumor cells were killed by PM21-NK cells to lesser extent as compared to SKOV3 cells expressing either NA-Fc3 or NA-Fc4 (18% vs 36% for NA-Fc3, and 18% vs 45% for NA-Fc4). For SKOV-3 cells expressing shorter versions of NA-Fc (NA1-2), increases in PM21-NK cell cytotoxicity did not reach statistical significance compared to naïve SKOV-3 cells. Taken together these data indicate that all four NA-Fc chimeras are expressed at the cell surface with an orientation that positions the Fc domain to mimic surface-bound IgG. Most importantly, ovarian tumor cells expressing two of the four chimeras with the longest NA stalk region (NA-Fc3 and 4) showed enhanced *in vitro* lysis by PM21-NK cells.

We extended this analysis of the NA-Fc chimeras to K562 myelogenous leukemia cells. As shown in S2 Fig, K562 cells showed a range of surface staining with antibody to Fc, with NA-Fc3 and -Fc4 having the lowest percent positive staining (Panel A) and lowest MFI (Panel B). In cytotoxicity assays with PM21-NK cells, K562 cells expressing NA-Fc4 had the highest level of killing after 20 mins at an E:T of 1:25 (Panel C) or 45 mins at an E:T of 1:0.625 (Panel D). Since NA-Fc4 was the most effective chimera in both of these cell lines, the remaining experiments below focused on this molecule.

### NA-Fc4 expression increases PM21-NK cell killing of ovarian and lung tumor cells in a real time assay

**Fig 3:**
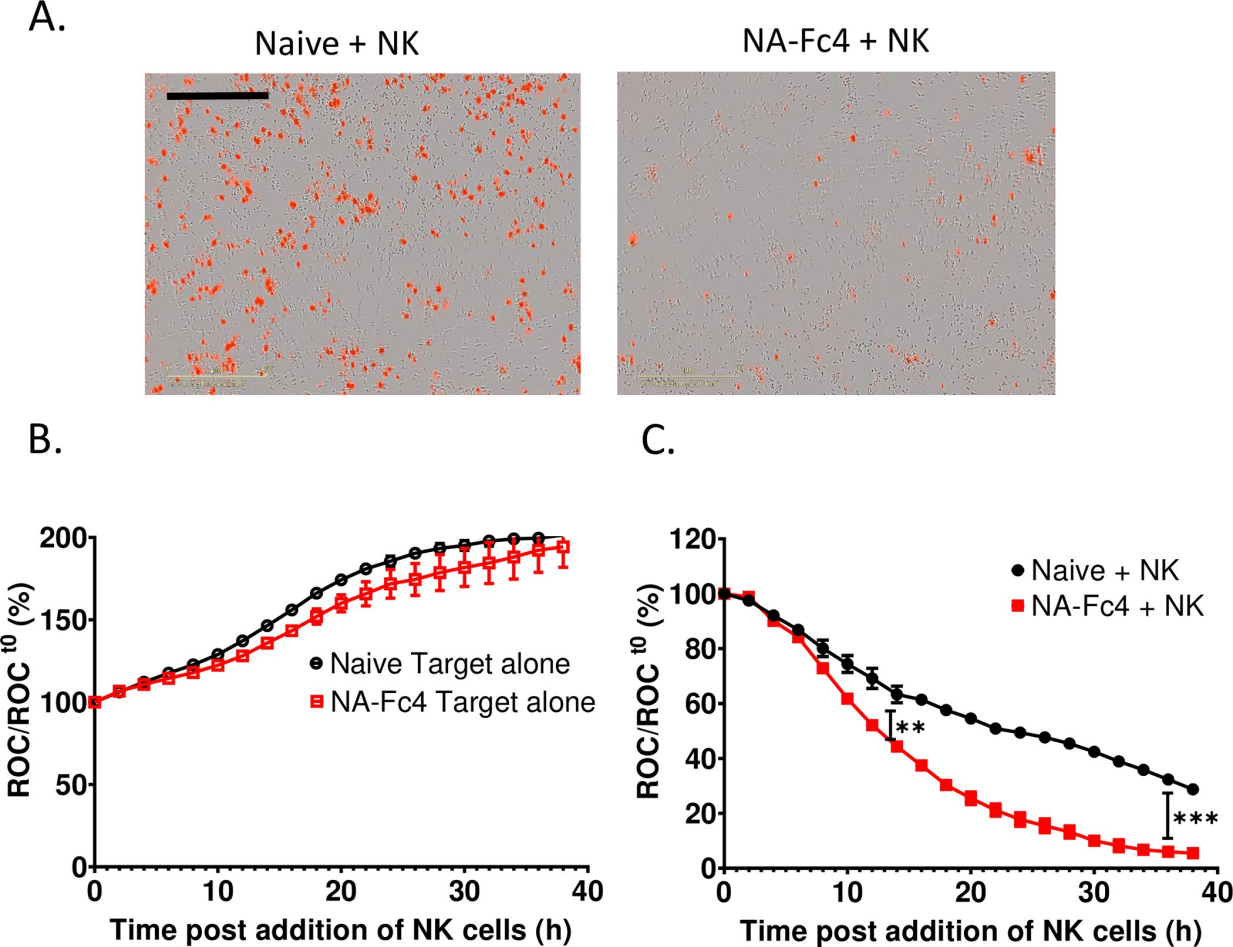
Real time assay for PM21-NK cell killing of NA-Fc4 ovarian cancer cells. SKOV3-NLR expressing NA-Fc4 were incubated with PM21-NK cells at an E:T ratio of 2.5:1. Red object count (ROC) was recorded on IncuCyte instrument at 2 h intervals and normalized to ROC at time 0 when PM21-NK cells were added to the target cells. (A) Phase and fluorescence images (10x) of naïve and NA-Fc4-expressing SKOV3 cultures at 30 h post addition of PM21-NK cells. The scale bar represents 400 μm. (B, C) Time dependent ROC curves of target cells + PM21-NK cells at an E:T ratio of 2.5:1. Panel B represents ROC of target alone samples; panel C represents ROC of indicated SKOV3 cells incubated with PM21-NK cells. Each data point represents an average of three samples with error bars as SD. Data was analyzed using two-way ANOVA test and by applying Dunett’s post hoc test when comparing with the control group. For all graphs, the adjusted p values after applying post hoc tests were *p<0.05, **p<0.01, ***p<0.001 when comparing naïve to NA-Fc cells. Stars indicate the point where the *p*-values first begins, and this statistical significance extends for later timepoints until a new star appears with a different p value.

To determine the kinetics of NA-Fc enhancement of PM21-NK cell mediated killing, we used SKOV3-NLR cells that possess red nuclei to which facilitates their detection in the IncuCyte instrument. This allowed real-time tracking and quantifying of killing of tumor cells by PM21-NK cells over a course of 30–40 h [32]. SKOV3-NLR cells were transduced with LV expressing NA-Fc4 and cells were bulk-sorted for high Fc expression using anti-Fc antibody as described in materials and methods section. Once sorted, the naïve and NA-Fc4 SKOV3-NLR cells were incubated with PM21-NK cells at an E:T of 2.5:1. The number of red nuclei (Red Object Count, ROC)) was recorded at 2 h intervals. Fig 3A shows an example of brightfield and fluorescence images captured at 24 h post addition of PM21-NK cells. Here, the images clearly show dramatic loss of red nuclei in the case of cells expressing NA-Fc4 + NK (right panel) as compared to the naïve cells + NK (left panel).

Data were collected overtime by the IncuCyte fluorescence detectors. At each timepoint, the data were expressed as remaining Red Object Count (ROC) normalized to the initial scan at time zero prior to addition of PM21-NK cells (ROC/ROC _t(0h)_). For both naïve and NA-Fc4 expressing SKOV3-NLR cells, Fig 3B shows timecourse curves for target cells incubated alone (target alone) without addition of PM21-NK cells. It is evident that the cells continue to proliferate, with ROC increasing up to ~200% of time 0 values. These data indicate that expression of NA-Fc4 chimera did not alter the growth or proliferative capacity of the cells. By contrast, the addition of PM21-NK cells to the NA-Fc4-expressing SKOV3-NLR cells (Fig 3, red curve, panel C) showed a more rapid decline in ROC, as compared to the naïve cells (Fig 3C, black curve) throughout the course of ~40 h. Together, these data from real-time killing assays show expression of the NA-Fc4 chimera on SKOV3-NLR tumor cells increases the rate and overall extent of killing by PM21-NK cells.

**Fig 4:**
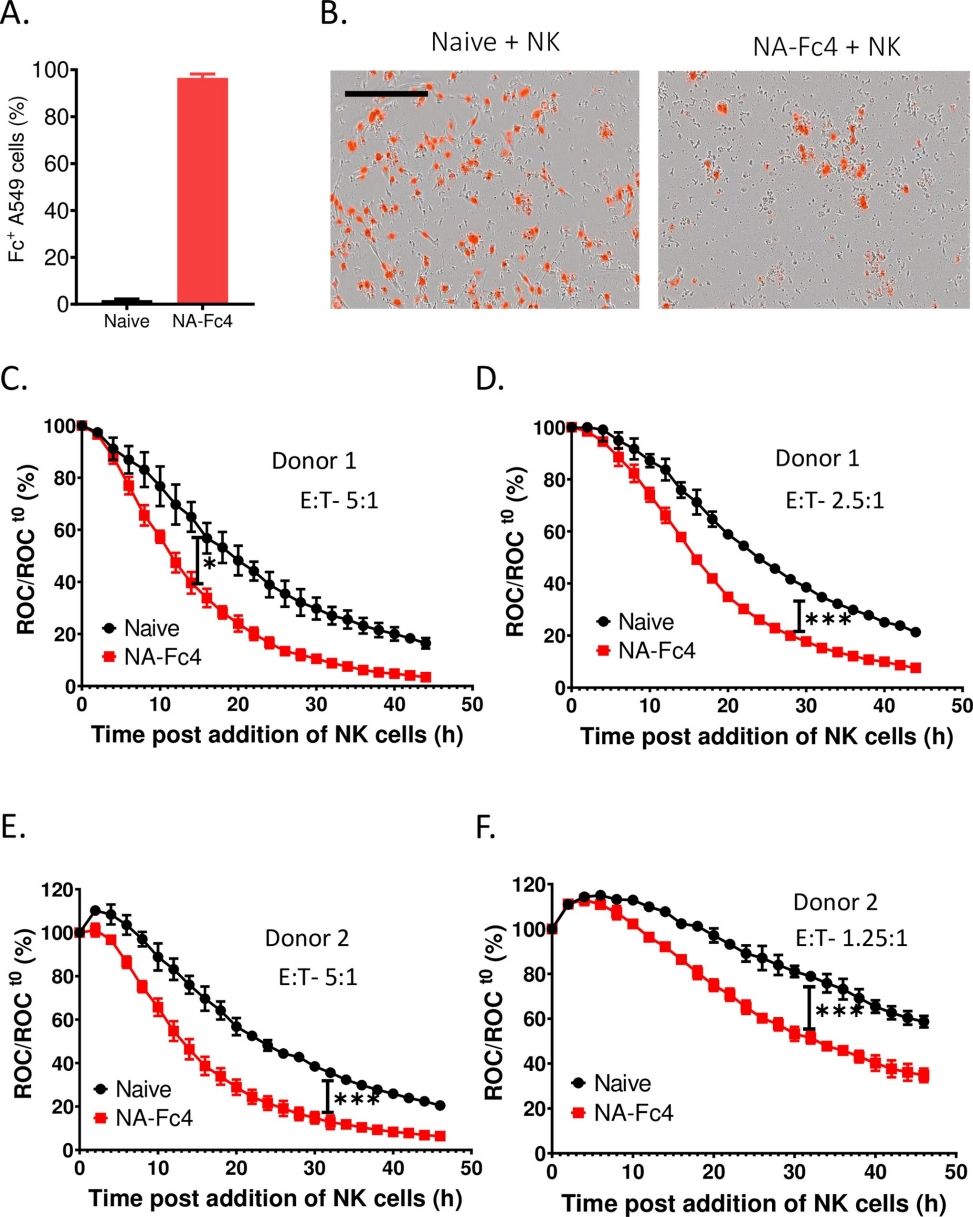
NA-Fc4 expression enhances PM21-NK cell killing of lung cancer cells. A549-NLR cells were transduced with LV encoding NA-Fc4 and sorted for stable NA-Fc expression. Cells were plated and incubated with PM21-NK cells at different E:T ratios. ROC was recorded on IncuCyte instrument at 2 h intervals and normalized to ROC at time 0 when PM21-NK cells were added to the target cells. (A) Fc expression of stable A549 NA-Fc4 NLR cells was quantified using flow cytometry post sorting. (B) PM21-NK cells were co-incubated with target cells, their phase and fluorescence images (10x) were captured for NK cell Donor 1 at 30 h post addition of PM21-NK cells. The scale bar represents 400μm. (C, D) Time dependent red intensity curves of target cells + PM21-NK cells were recorded at an interval of 2 hrs at two different E:T ratios, 5:1 and 2.5:1 respectively. Panels (E, F) represent normalized ROC for naïve and NA-Fc4 cells when incubated with Donor 2 at two different E:T ratios of 5:1 and 1.25:1. Each data point represents an average of three samples with error bars as SD. Data was analyzed using two-way ANOVA test and by applying Dunett’s post hoc test when comparing with the control groups. For all graphs, the adjusted p values after applying post hoc tests were *p<0.05, **p<0.01, ***p<0.001. Stars indicate the point where the *p*-values first begin, and this statistical significance extends for later timepoints until a new star appears with a different p value.

To test if NA-Fc expression enhances NK cell killing in other cell types, the same assay was performed with A549 non-small cell lung cancer cells. A549-NLR cells were transduced with LV expressing NA-Fc4 and cells were sorted for Fc expression and stained with antibody to Fc. As shown in Fig 4A, 95–98% of cells stained positive for Fc expression. PM21-NK cells were incubated with naïve or A549-NLR cell expressing NA-Fc4 chimera at several E:T ratios. Fig 4B shows a snapshot of image captured on IncuCyte at 30 h post incubation with PM21-NK cells for donor 1 at a ratio of 2.5:1, where it is evident that the NA-Fc expressing cells (Right panel) are more extensively killed compared to naïve cells (left panel). Timecourses of reduction in ROC are shown in Fig 4C–4F at different E:T ratios and with different donor NK cells. Incubation of PM21-NK cells with A549-NLR cells expressing NA-Fc4 resulted in enhanced killing of target cells compared to naïve A549-NLR cells in case of both NK cell donor 1 (Fig 4C and 4D) and donor 2 (E, F). Taken together, these data show that expression of NA-Fc4 chimera on human lung cancer cells increases their susceptibility to PM21-NK cell killing in vitro.

### NA-Fc4 expression on target cells leads to increases in PM21-NK cell degranulation and cytokine release

**Fig 5:**
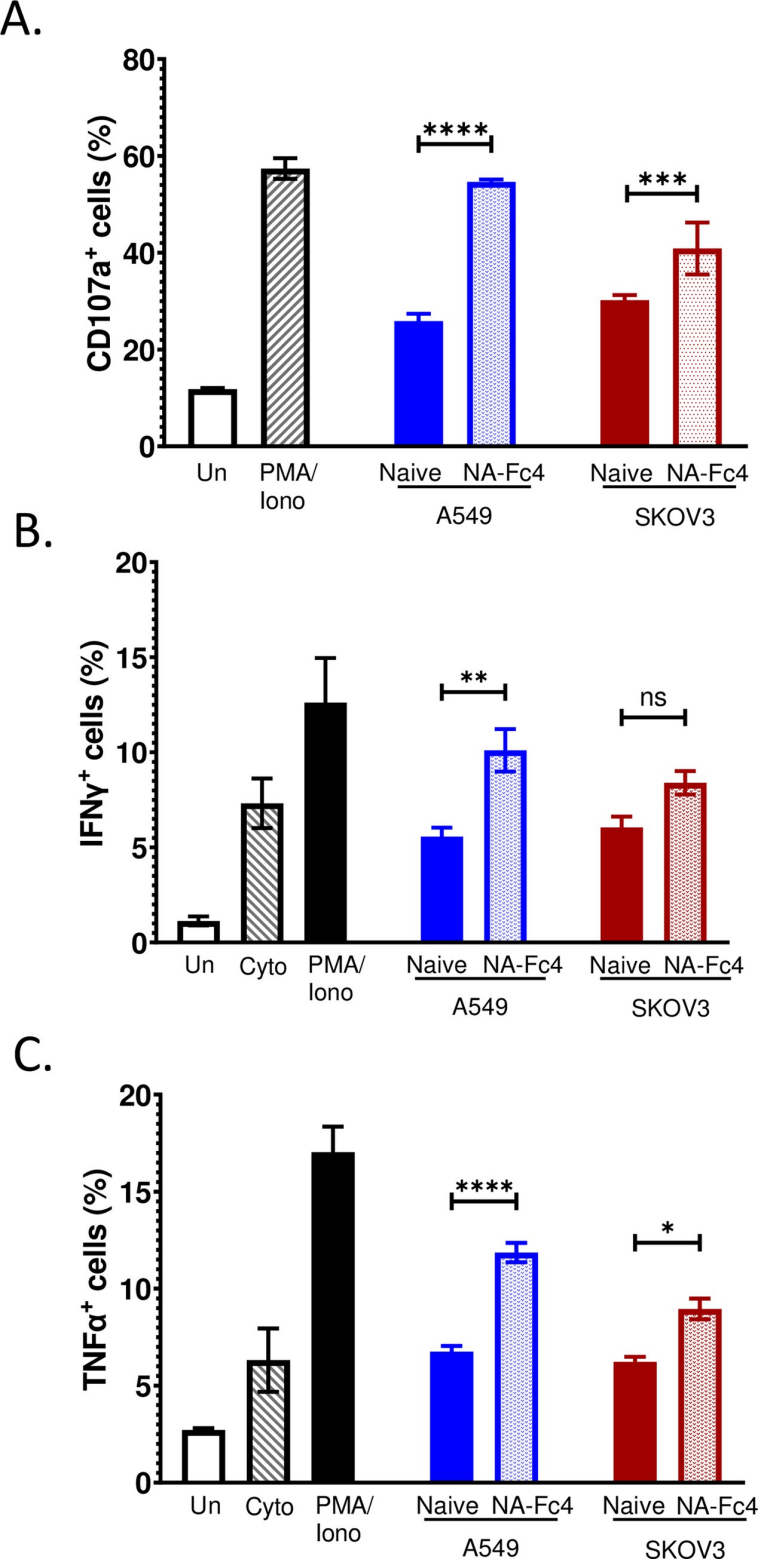
NA-Fc4 expression on target tumor cells enhances NK cell activity. PM21-NK cells were incubated for 16 h with indicated naïve or NA-Fc4 expressing target cells at a 1:1 ratio. As controls, cells were left untreated or treated with PMA/Ionomycin drugs or a cocktail of cytokines known to induce NK cell cytokine secretion (Cyto). A) PM21-NK cells were then assessed by flow cytometry for cell surface CD107a expression as a measure of degranulation, or alternatively stained intracellularly for IFN-γ (panel B) or TNF-α (panel C). Values are a mean of three samples with error bars as SD. Data was analyzed using two-way ANOVA analysis. For all graphs *p<0.05, **p<0.01, ***p<0.001 and ****p<0.0001 when comparing indicated groups of naïve and NA-Fc4.

Engagement of CD16 receptor on NK cells with Fc portion of antibody leads to degranulation, along with cell surface expression of CD107a and release of cytokines such as TNF-α and IFN-γ [14,17], all of which correlates with enhanced NK cell activity [16]. To determine if NA-Fc expression on target cells leads to increases in these markers of PM21-NK cell activity, naïve A549-NLR or SKOV3 cells or their corresponding NA-Fc-expressing cell lines were incubated with PM21-NK cells at an E:T ratio of 1:1 for 16 h. As a positive control, PM21-NK cells were treated either with drugs PMA/Ionomycin or a cocktail of cytokines of IL12/IL15/IL18. PM21-NK cells were assessed for surface expression of CD107a via flow cytometry. As shown in Fig 5A, there was an increase in the percentage of PM21-NK cells with high CD107 expression when co-cultured with NA-Fc expressing cells compared to control cells. Interestingly, the amount of CD107a expressed on NK cells when incubated with A549 NA-Fc4 was similar to the amount expressed on NK cells when incubated with the positive control PMA/Ionomycin (black bars).

Similar results were seen when PM21-NK cells were assayed for intracellular TNF-α and IFN-γ expression by flow cytometry. As shown in Fig 5B and 5C, NK cells incubated with target cancer cells expressing NA-Fc4 had an increase over control cells in TNF-α and IFN-γ staining in the case of A549 cells but only in TNF-α staining for SKOV3 cells. Taken together, these data indicate that NA-Fc4 expression on tumor cells increases the activity of PM21-NK cells.

### NA-Fc4 mediated enhancement of PM21-NK cell killing involves Fc and CD16 engagement

**Fig 6:**
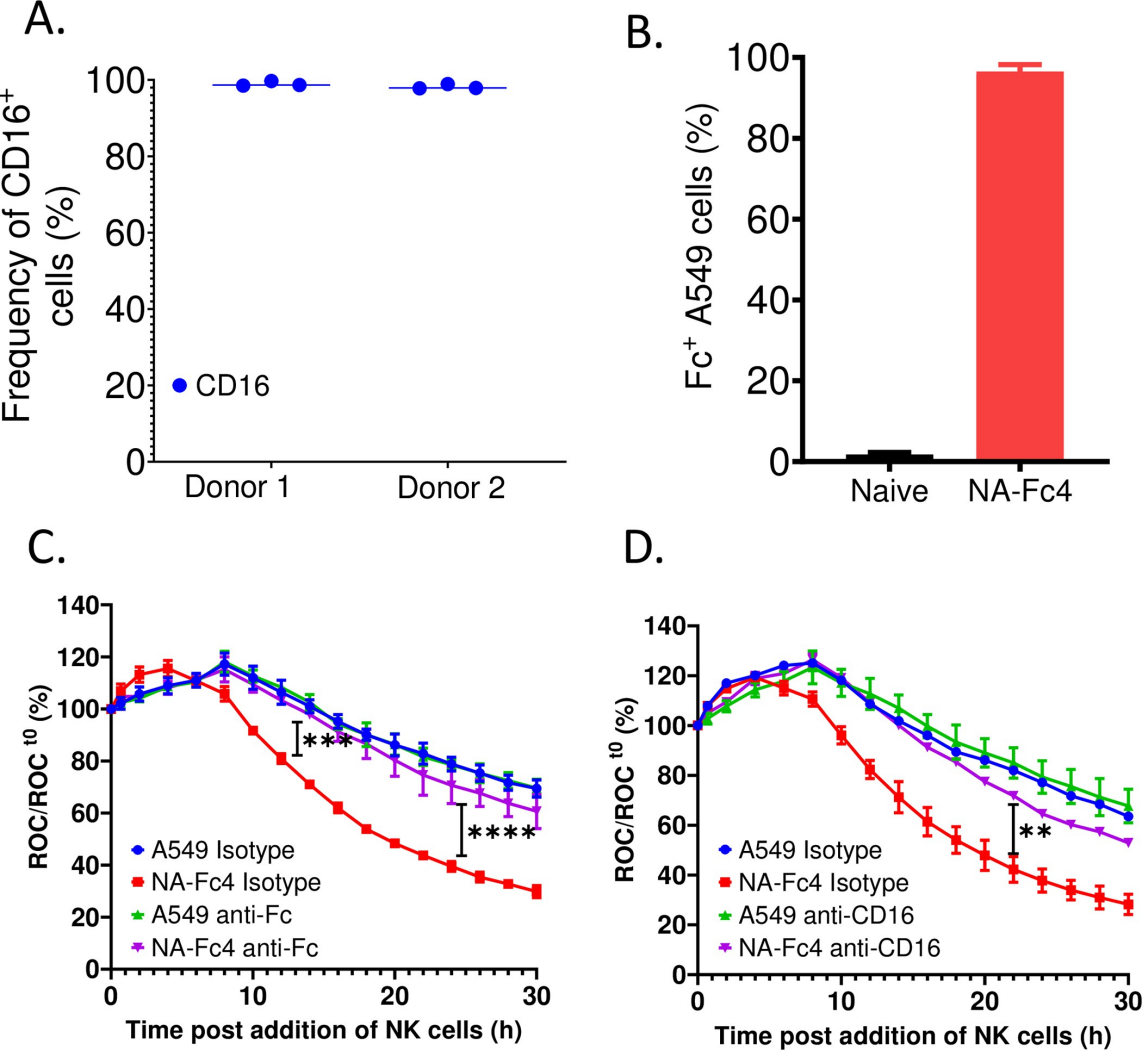
NA-Fc4 mediated enhancement of PM21-NK cell killing requires engagement of CD16 receptor and Fc ligand. (A) NK cells from donors 1 or 2 were stained with antibodies to CD56 and CD16. They were gated for CD56 to determine the percent of NK cell population expressing CD16. (B) Target A549-NLR cells were stained with antibody to IgG1 Fc domain to quantify surface Fc expression. (C) Target naïve A549-NLR cells or NA-Fc4 expressing cells were treated with Fc blocking antibody or isotype control antibody prior to incubation with PM21-NK cells at an E:T ratio of 5:1. (D) Similarly, PM21-NK cells were treated with anti-CD16 antibody or isotype control prior to incubation with either of the target cells at an E:T of 5:1. For both experiments, ROC was recorded every 2 h and images were captured on IncuCyte. Values are a mean of 3 replicates and error bars represent SD. Data was analyzed using two-way ANOVA test and by applying Tukey’s post hoc test when comparing with the indicated groups. For all graphs, the adjusted p values after applying post hoc tests were **p<0.01, ***p<0.001 and ****p<0.001. Stars indicate the point where the *p*-values first begin, and this statistical significance extends for later timepoints until a new star appears with a different p value.

ADCC is activated by engagement of the CD16 receptor on NK cells with Fc portion of antibodies on the surface of target cells [4,33]. PM21-NK cells from two donors (1 and 2) were stained using antibodies to CD16 and CD56 (an NK cell marker) and analyzed by flow cytometry. As shown in Fig 6A, a vast majority of PM21-NK cells expressed high levels of CD16.

Blocking experiments were performed to determine if enhancement of NK cell killing of NA-Fc-expressing target cells is dependent on CD16 on NK cells. Target naïve A549-NLR cells and A549-NLR cells expressing NA-Fc4 were first pretreated with Fc blocking antibody (to block Fc ligand on target cells) or isotype control for 1 h and then co-incubated with PM21-NK cells at an E:T ratio of 5:1. When analyzed on IncuCyte instrument, treatment of NA-Fc4-expressing cells with Fc antibody (Fig 6C, purple curve) resulted in significant reduction in PM21-NK-mediated killing of tumor cells as compared to the isotype control (Fig 6C, red curve). Antibody pre-treatment of naïve A549-NLR cells (Fig 6C; blue curve compared with green curve) did not alter PM21-NK cell killing.

The role of CD16 in PM21-NK cell-mediated killing of NA-Fc4 cells was tested by pretreating PM21-NK cells with anti-CD16 or isotype control antibody and then co-incubating with naïve or NA-Fc4-expressing A549-NLR cells. Killing was assessed on IncuCyte instrument and ROC was recorded every 2 h. As shown in Fig 6D, PM21-NK cells treated with isotype control antibody effective killed cells expressing NA-Fc4 (red curve). Importantly, the kinetics and extent of PM21-NK cell killing were reduced by treatment with CD16 antibody (purple curve). Anti-CD16 treatment of PM21-NK cells had a negligible effect on killing of naïve A549-NLR that did not express NA-Fc (blue and green curves). Together, these data support the contention that NA-Fc expression on target cells mimics the natural Fc-CD16 engagement and enhances NK cell killing similarly to ADCC.

### NA-Fc4 expression does not drive complement-mediated lysis of cancer cell lines

**Fig 7:**
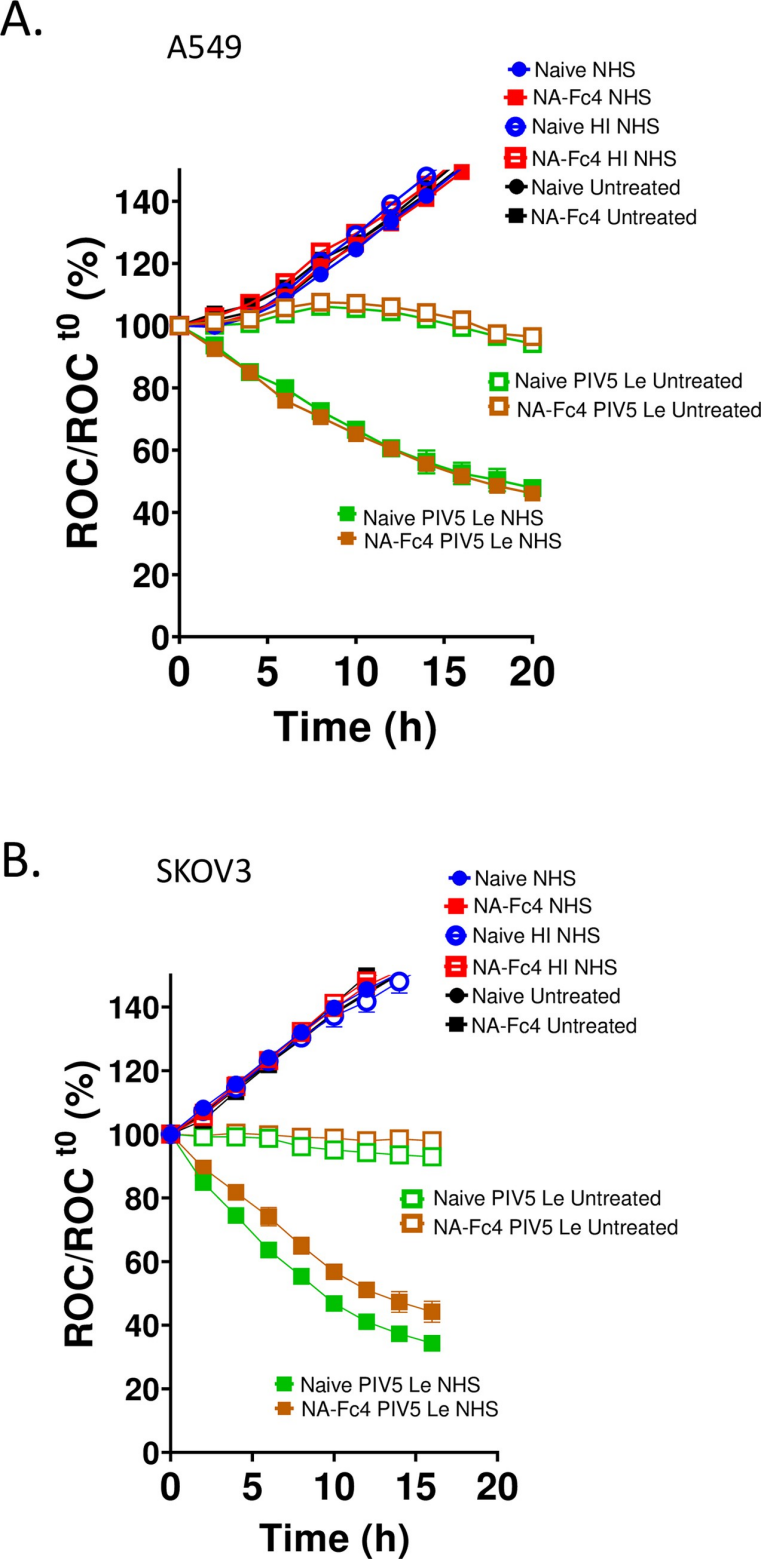
Complement-mediated lysis of tumor cell lines is not enhanced by NA-Fc4 expression. Naïve A549-NLR (panel A) and SKOV3-NLR (panel B) cells as well the corresponding cell lines that stably express NA-Fc4 were left untreated or incubated with 20% normal human serum (NHS) or heat-inactivated NHS (HI). As positive controls for complement lysis, cells were alternatively infected with PIV5 and treated as above. ROC was recorded every 2 h on IncuCyte.

To test the ability of NA-Fc to engage complement and drive complement mediated lysis of tumor cells, target naïve or NA-Fc expressing A549-NLR cells were treated with NHS (source of complement), HI NHS (complement-deficient) or left untreated. As a positive control, both target cells were infected with parainfluenza virus type 5 (PIV5), which we have shown in our published work [34] to be a strong activator of complement-mediated lysis (solid green curves panel). As shown in Fig 7, A549 cells expressing NA-Fc did not succumb to complement mediated lysis (solid square red curve). Similar results were seen with SKOV3 ovarian cancer cells expressing NA-Fc, which were not sensitized to complement-mediated killing compared to naïve control cells (Fig 7).

### Lentivirus delivery of NA-Fc sensitizes tumor cells to enhanced PM21-NK cell-mediated killing

**Fig 8:**
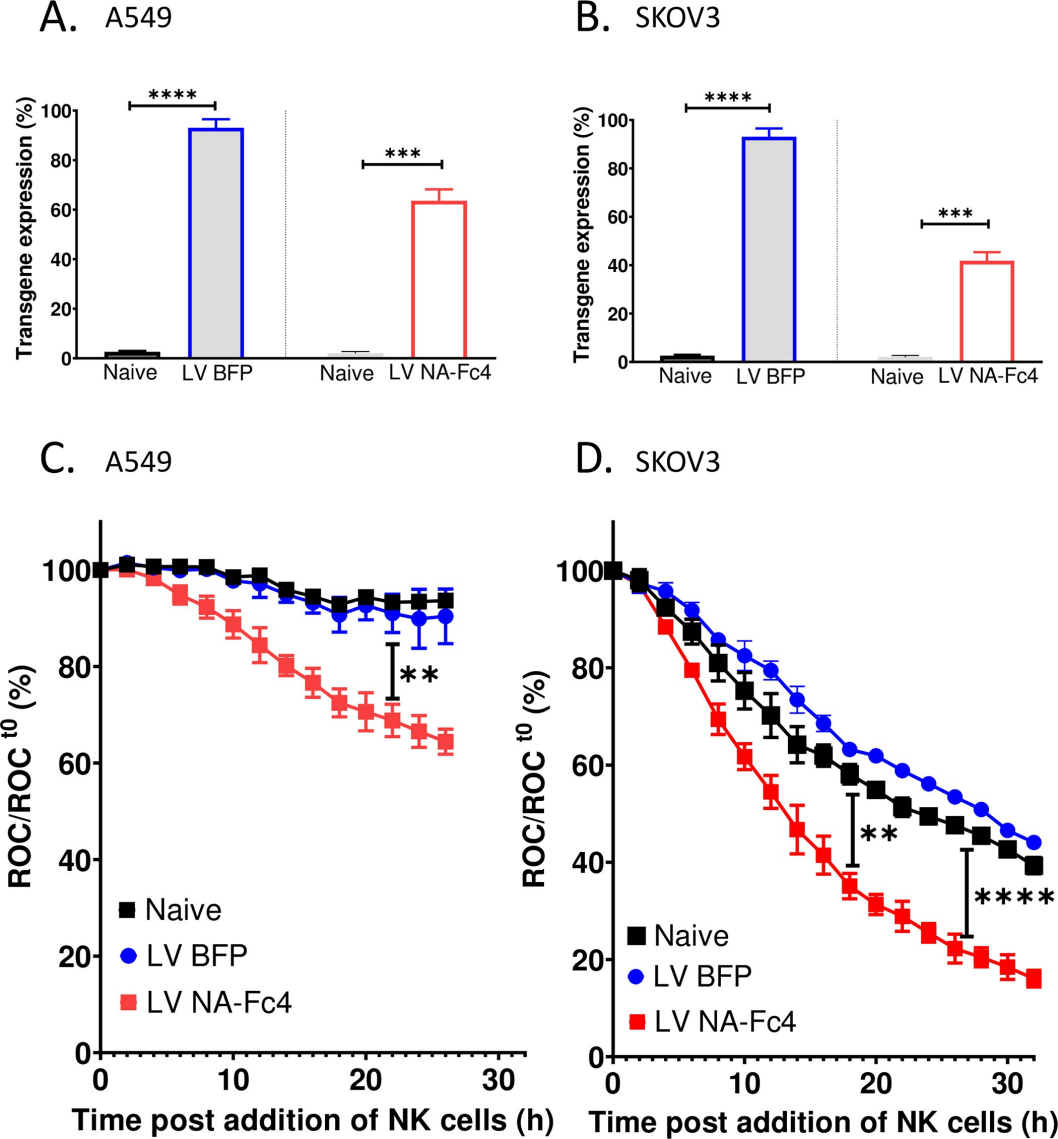
Lentiviral delivery of NA-Fc4 enhances the susceptibility of naïve A549 lung and SKOV3 ovarian tumor cells to PM21-NK cell mediated lysis. (A, B) A549-NLR and SKOV3-NLR cells were transduced with LV expressing either NA-Fc4 or BFP as a control. Two days post transduction, transgene expression was quantified using flow cytometry by staining all the samples with IgG Fc. (C, D) The naïve target cells or cells transduced with LV-BFP or LV NA-Fc4 were co-incubated with PM21-NK cells at an E:T ratio of 2.5:1. ROC was recorded every 2 h on IncuCyte. Results are representative of two independent experiments conducted with two different NK cell donors in triplicate with error bars representing SD. Data was analyzed using one-way ANOVA & two-way ANOVA test and by applying Dunett’s post hoc test when comparing the indicated control group. For all graphs, adjusted p values after applying post hoc tests were **p<0.01, ***p<0.001 and ****p<0.0001. Stars indicate the point where the *p*-values first begin, and this statistical significance extends for later timepoints until a new star appears with a different p value.

For therapeutic utility, NA-Fc molecules would need to be selectively delivered to tumors by oncolytic viruses to sensitize them to killing by adoptively transferred NK cells. To test this as proof of concept of viral delivery, we expressed NA-Fc4 or the control blue fluorescence protein (BFP) through lentiviral (LV) delivery to NLR-expressing cells followed by incubation with PM21-NK cells and assessed the killing by IncuCyte assays. As shown in Fig 8, transduction of A549-NLR (panel A) or SKOV3-NLR (panel B) cells with LV expressing NA-Fc4 or a control blue florescence protein (BFP), showed ~80–90% of cells expressing BFP (grey filled blue bars) and ~40–45% of cells expressing surface Fc protein (hollow red bars) by 2 d post transduction (2 dpt). The control naïve cells showed negligible positive staining for BFP or NA-Fc4 (when stained with Fc antibody). As shown in Fig 8C, when these transduced cells were co-incubated with PM21-NK cells, lung tumor cells transduced with LV NA-Fc4 (red curves) showed a more rapid and extensive reduction in ROC as compared to the cells transduced with control LV BFP (blue curves). These results were also seen for ovarian cancer cells (Fig 8D). Control untreated naïve cells (solid black curve) were killed by NK cells at a similar rate as the cells transduced with LV BFP (Fig 8C and 8D), supporting the contention that viral delivery of NA-Fc will result in enhanced killing by NK cells.

**Fig 9:**
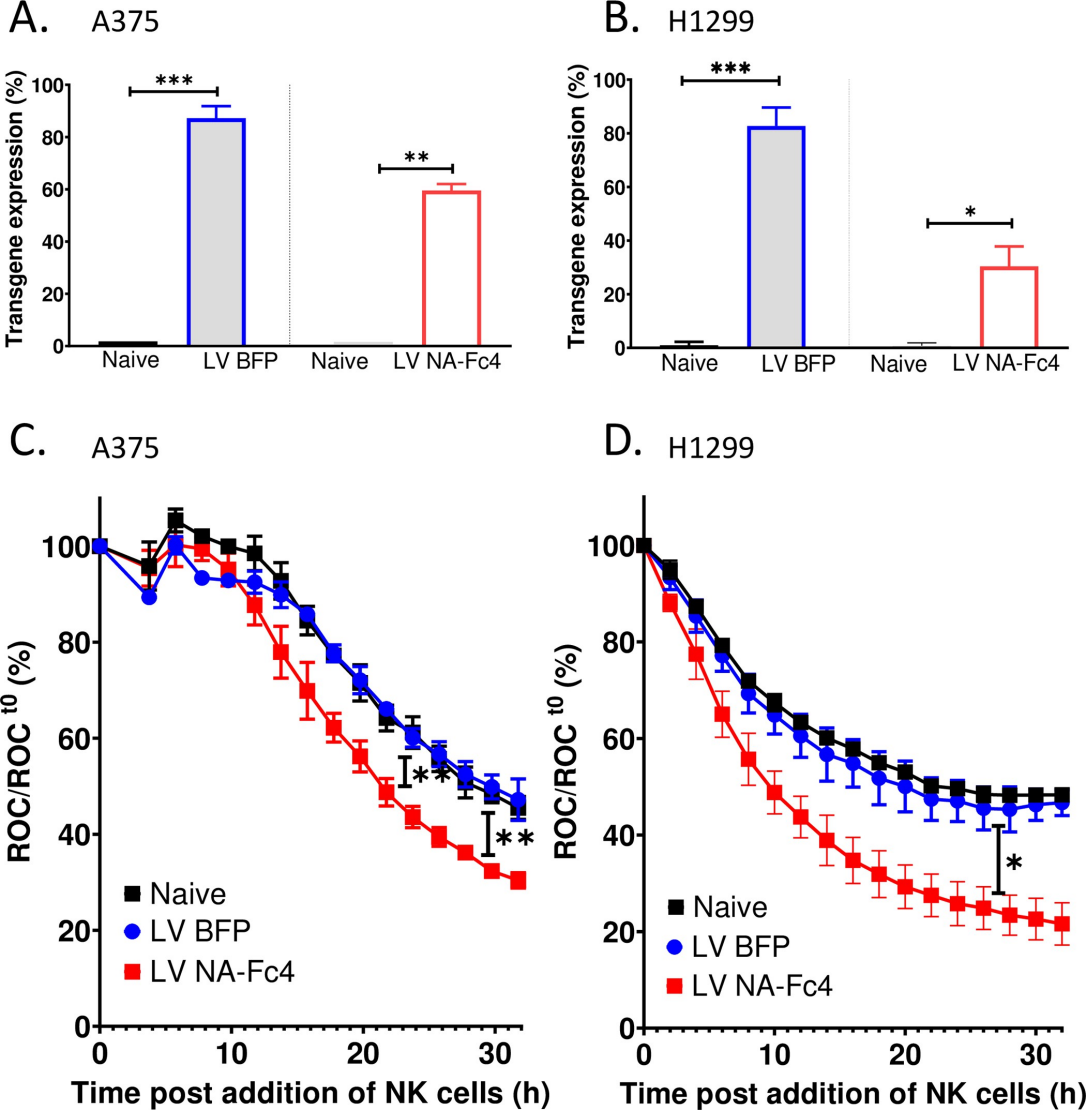
Lentiviral delivery of Fc protein enhances the susceptibility of naïve A375 melanoma and H1299 lung tumor cells to PM21-NK cell mediated lysis. (A, B) A375-NLR and H1299-NLR cells were transduced with LV expressing NA-Fc4 or BFP as a control. Two days post transduction, transgene expression was quantified using flow cytometer by staining all the samples with IgG Fc antibody. (C, D) The target cells naïve, LV BFP or LV NA-Fc4 were co-incubated with PM21-NK cells at an E:T ratio of 2.5:1. ROC was recorded every 2 h on IncuCyte. These experiments are a representative of two independent experiments conducted with two different NK cell donors in triplicates with error bars representing SD. Data was analyzed using one-way ANOVA & two-way ANOVA test and by applying Dunett’s post hoc test when comparing with the indicated control group. For all graphs, the adjusted p values after applying post hoc tests were *p<0.05, **p<0.01 and ***p<0.001. Stars indicate the point where the *p*-values first begin, and this statistical significance extends for later timepoints until a new star appears with a different p value.

Similar experiments were performed on A375-NLR melanoma cancer cells and H1299-NLR lung cancer cells which were transduced with LVs expressing either BFP or NA-Fc4 or were left untreated. As shown in Fig 9, BFP was expressed in ~80–90% of both H1299 and A375 cells, whereas NA-Fc4 expression was ~60% in case of A375 cells and ~25% for H1299 cells (panels C and D). Despite the low transduction efficiency, co-culturing with PM21-NK cells resulted in a rapid and extensive decline in ROC for cells expressing NA-Fc4 (red closed squares, Fig 9) as compared to the naïve (solid square black curve) or LV BFP (solid circle blue curve). The data in Figs 8 and 9 demonstrate that expression of NA-Fc4 in four different tumor cell lines conferred increased sensitivity to killing by PM21-NK cells.

### Delivery of NA-Fc to parainfluenza virus-persistently infected cells overcomes their resistance to PM21-NK cell mediated killing

**Fig 10:**
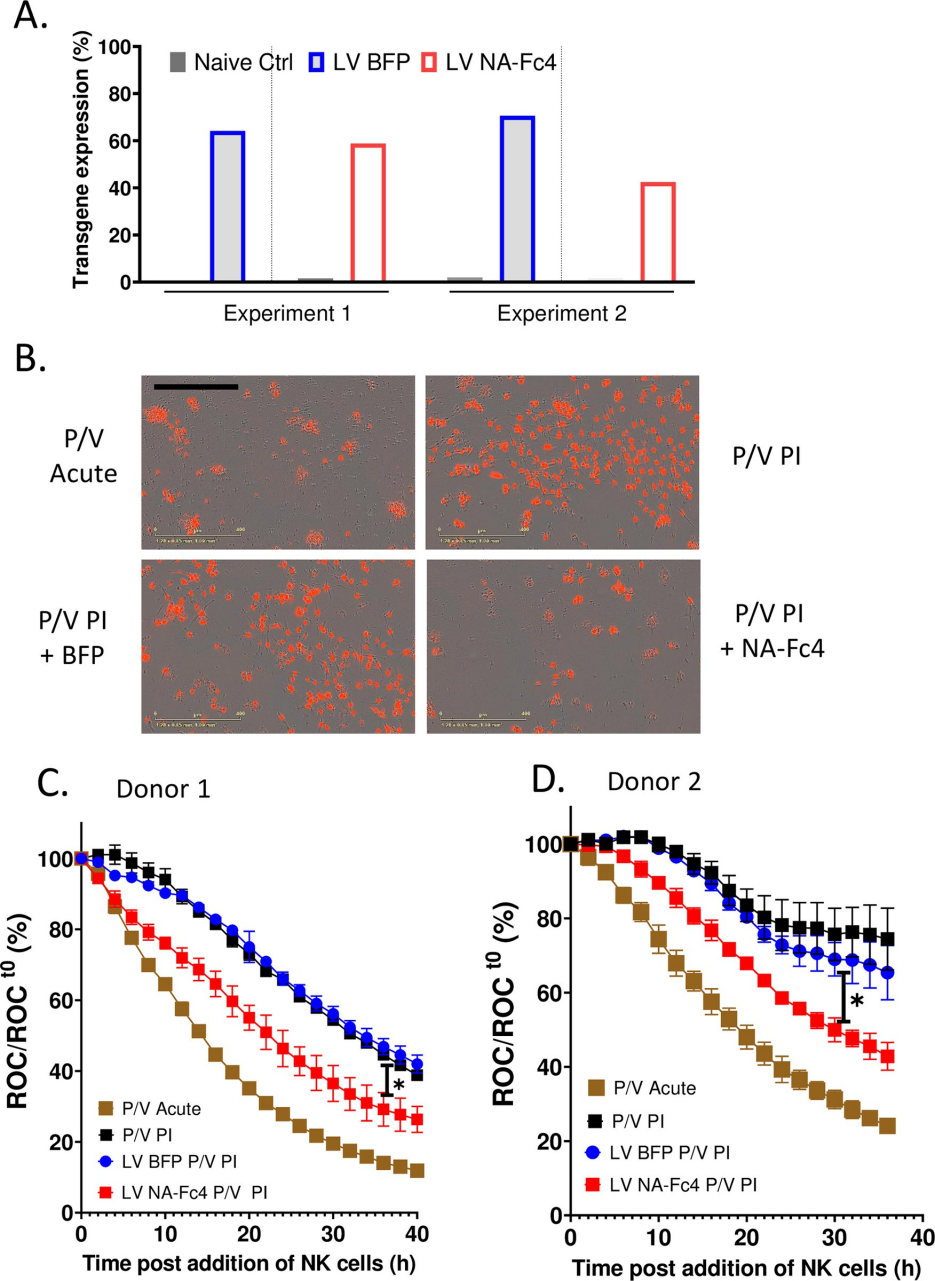
Lentiviral delivery of NA-Fc4 enhances PM21-NK cell mediated killing of lung cells persistently infected with PIV5 mutant virus. (A) P/V PI-NLR A549 cells were transduced with LV expressing NA-Fc4 or BFP as a control. Two days post transduction, transgene expression was quantified using flow cytometry. The untreated P/V PI cells stained with Fc antibody were used as controls. (B-D) The target A549 cells were acutely infected with P/V mutant, persistently infected A549 cells (P/V PI), P/V PI transduced with LV BFP or LV NA-Fc4 were plated and co-incubated with PM21-NK cells at an E:T ratio of 1.25:1. ROC was recorded every 2 h on IncuCyte. (B) Phase and red fluorescent images (10x) were captured at 32 h post addition of NK cells donor 2. The scale bar represents 400μm. (C, D) ROC were recorded on IncuCyte when each of the samples were co-incubated with NK cells Donor 1 and Donor 2. Curves represent data for acutely-infected cells (brown), P/V PI cells (black), or P/V PI cells transduced to express BFP (blue) or NA-Fc4 (red). Each data point is an average of 3 replicate samples with error bars representing SD. Data was analyzed using two-way ANOVA test and by applying Dunett’s post hoc test when comparing with the indicated groups. For both the graphs, the adjusted p values after applying post hoc tests were *p<0.05. Stars indicate the point where the *p*-values first begin, and this statistical significance extends for later timepoints until a new star appears with a different p value.

Many RNA viruses can establish persistent infections (PI) of cells, whereby virus continues to replicate and persist in the cytoplasm, but for unknown reasons, the cells are resistant to killing by many immune mechanisms such as NK cells. We have previously shown that a parainfluenza virus (PIV5) termed P/V virus can establish a PI in some human lung cancer cell lines [35]. We tested the hypothesis that delivery of NA-Fc4 to these PI cells would enhance their killing by PM21-NK cells. The previously described A549-NLR cells which are PI with the P/V virus [32,35] were transduced with LVs expressing BFP or NA-Fc4 or left untreated. As shown in Fig 10A, transgene expression in two independent experiments showed that the percentage of cells expressing NA-Fc (red bars) ranged from ~40–60% whereas BFP expression (grey filled blue bars) was in ~60–70% of cells. The control naïve cells showed negligible positive staining for BFP or NA-Fc4 (when stained with Fc antibody).

Cells were co-cultured with PM21-NK cells at an E:T of 1.25:1 from two independent donors and killing was assayed by IncuCyte. Fig 10B shows snapshots of brightfield and fluorescence images captured at 32 h post addition of PM21-NK cells. A549-NLR cells acutely infected with P/V virus (upper left panel) were killed to a large extent by PM21-NK cells as shown our prior published work [32]. By contrast, more red nuclei (intact target cells) are evident in case of PM-21 NK cells incubated with A549-NLR cells that harbor the P/V PI (upper right panel), consistent with the interpretation that PI cells are more resistant to NK cell killing. As shown in Fig 10B (lower right panel), LV delivery of NA-Fc4 to the P/V PI cells followed by incubation with PM21-NK cells resulted in greater loss of red nuclei (lower right panel) as compared to the control BFP LV transduced cells (lower left panel).

The kinetics and extent of PM21-NK cell killing of P/V PI cells was quantified by IncuCyte real time assays using NK cells from two different donors. Fig 10C and 10D show changes in ROC counts over time for cells that were either acutely or PI with the P/V virus and transduced with either BFP control or NA-Fc. As was previously reported [32], A549-NLR cells acutely infected with P/V virus were efficiently killed by PM21-NK cells (brown boxes), but this was not seen in the case of A549-NLR cells harboring the P/V PI (black boxes). Transduction of the P/V PI cells to express BFP had no effect on NK cell killing (blue curves). Most importantly, treatment of P/V PI cells that expressed NA-Fc with PM21-NK cells (red curve) resulted in more rapid decline in the ROC as compared to the controls naïve targets (black squares). These data support potential use of NA-Fc to aid in NK cell-mediated elimination of persistent infections.

## Discussion

ADCC is one of the most powerful mechanisms for NK cells to kill cancer and virus-infected cells. This has sparked intense interest in pre-clinical studies to discover novel antibody-related approaches to enhance Fc-receptor mediated functionalities in ADCC. However, traditional approaches to ADCC rely on developing antibodies with desired properties and prior knowledge of the antigens to be targeted by ADCC. Here we have shown proof-of-concept on an approach to deliver a novel membrane-anchored fusion protein which can mark tumor cells with strong signals to promote NK cell killing irrespective of pre-existing antibodies to known antigens. Our results support the further development of this approach to express a functional Fc domain on the surface of tumor cells and virus infected cells, leading to an increased NK cell-mediated killing of these target cells. These results are presented in the context of immunotherapeutic approaches that use adoptive transfer of NK cells, with the future goal of combining NK cells with tumor-specific oncolytic viruses that express NA-Fc to enhance tumor cell killing by both enhancing NK cell killing and preventing a potential persistent infection to allow complete cancer clearance.

Our analysis showed that NA-Fc chimeras with differing lengths of the stalk region which extends Fc from the plasma membrane had differing levels of cell surface expression, consistent with prior work on proteins with truncated lengths being effectively transported [29,30]. Most importantly, the chimeric NA-Fc with the longest spacer region was expressed at the cell surface at the lowest steady state level of our four chimeras, and yet was the strongest activator of target cell killing when exposed to PM21-NK cells. This could reflect a need for optimal spatial alignment and distance from the membrane of the Fc domain on target cells to interact properly with NK cell receptors such as CD16. Alternatively, other properties of the different chimeric molecules could impact their ability to stimulate cytotoxicity, including extent of glycosylation, oligomerization, and distribution in plasma membrane lipid rafts. The panel of NA-Fc chimera could provide a foundation for systematic analyses of these factors. These data support the contention that levels of cell surface expression are not necessarily the only major driver of which molecules activate NK cell-mediated killing of cancer cells.

Over the years there have been continuous improvements in generation of mAbs which could enhance ADCC [4,6,33,36–38]. There are a number of phagocytic cell types that can recognize antibody-coated target cells, including macrophages and NK cells. In the context of adoptive immunotherapy, NK cells can play a crucial role in mediating anti-tumor activity along with therapeutic mAbs, including examples such as rituximab [39], trastuzimab [40,41], cetuximab [42] or isatuximab [43]. Several studies have shown that the overall effect of an antibody on ADCC can be improved by introduction of enhancing amino acid changes in the Fc domain or by introducing mutations in the CD16 receptor of NK cells [9,37,44]. We are in the process of testing new versions of our NA-Fc molecule to include these Fc alterations for improved efficacy in PM21-NK cell killing.

Our *in vitro* analysis of cells expressing NA-Fc show that complement-mediated lysis was not increased compared to naïve control cells, both with A549 lung tumor cells and SKOV3 ovarian cancer cells. The reasons why a surface-bound Fc molecule could enhance killing by PM21-NK cells but not by complement are not known at this point. Possible limitations on complement-mediated killing could include expression of complement inhibitors, expression levels that are below a threshold for complement activation, or the spatial orientation of the chimeric NA-Fc molecule with relation to the plasma membrane and/or other surface proteins.

Some viral infections proceed from an acute phase to a persistent phase, whereby infected cells continue to survive with ongoing viral replication. We have previously shown that an oncolytic Parainfluenza virus 5 containing mutations in the P/V gene can cause a PI in human lung cancer cell lines [35], similar to that seen with certain other oncolytic viruses [45–49]. This is a potential risk associated with use of OV that can also result in tumor escape and regrowth. Here, we show that cells acutely infected with the P/V virus are killed more rapidly by PM21-NK cells as described in our prior work [32]. Most importantly, cells which harbor a PI with the P/V virus are killed much less effectively *in vitro* by PM21-NK cells, for reasons that we currently do not understand. This finding indicates that the transition from P/V virus acute infection to PI leads to changes in the cells which reduce their susceptibility to NK cell lysis and potentially immune-mediated elimination. Future work will define whether these changes involve loss of positive-acting signals or increase in negative-acting inhibitory signaling. Our finding reported here that PM21-NK cell killing is improved when the NA-Fc molecule is delivered to the PI cells indicates that this approach can potentially improve the efficacy of oncolytic virotherapy by enhancing clearance of resistant tumor cells, including PI cells to lower the chance of relapse.

Here we have used lentiviruses as the vector for our proof-of-principle that NA-Fc could be delivered to cancer or virus-infected cells to improve NK cell-mediated killing. However, a long-term goal would be to use oncolytic viruses to deliver the NA-Fc in conjunction with adoptive NK cells to combine the powerful cancer cell killing properties of an oncolytic virus with enhanced elimination by adoptively transferred, donor-derived, hyperactive NK cells such as PM21-NK cells. This raises the question of which oncolytic virus would be appropriate for delivery of NA-Fc, since the signals on virus-infected cells for NK cell killing (e.g. surface glycoproteins) could act to mask any contributions of NA-Fc. Some studies have demonstrated the concept of delivering Fc-fusion proteins with the help of oncolytic viruses such as SIRPα-Fc fusion protein against ovarian cancer [50], expression of antibody in HSV-1 oncolytic virus against glioblastoma [51], and adenovirus against breast cancer in mouse model [52].

## Conclusions

Taken together our results demonstrate expression of a novel membrane-anchored Fc-containing molecule which is oriented at the plasma membrane similar to an exogenous antibody would be when bound to the cell surface. This NA-Fc molecule was capable of conveying high level NK cell killing in the case of 5 different cell lines, through interactions between CD16 and the target cell Fc domain. The delivery of NA-Fc also conveyed increased NK cell killing of cells that were persistently infected with parainfluenza virus, raising the proposal that this approach could be used as a general strategy to enhance Fc-driven killing of both cancer cells and virus infected cells. Future work will be focused on mechanisms to deliver this novel molecule to cancer cells and in vivo studies, including the use of OV vectors to improve the selectivity of NK cell-mediated killing.

## Supporting information

S1 Fig Example of gating strategy for measuring CD56+ NK cells. (TIF)

S2 Fig NA-Fc4 enhances PM21-NK cell killing of K562 myelogenous leukemia cells. Parental K562 cells and K562 cells expressing one of the four NA-Fc chimeras were stained with stained with antibody to Fc domain. Percent positive (panel A) and MFI (Panel B) for surface Fc expression was assayed by flow cytometry. PM21-NK cells were incubated with cultures of the indicated K562 cells for 20 mins at an E:T of 1.25:1(Panel C) or 45 mins at an E:T of 0.625:1 (Panel D). Percent cytotoxicity was determined using a flow based cytometric assay as described in Material & Methods. Data is from triplicate samples with error bars represent SD. Data was analyzed using two-way ANOVA analysis. * and ** indicate p values of p<0.05 and p<0.01.
(PDF)

S3 Fig SKOV3 Naive and NAFc4 target only Fig 3B. (PDF)

S4 Fig SKOV3 Naive and NAFc4 plus NK Fig 3C. (PDF)

S5 Fig A549 Naive and NAFc4 plus Donor 1 NK 5 Fig 4C. (PDF)

S6 Fig A549 Naive and NAFc4 plus Donor 1 NK 2.5 Fig 4D. (PDF)

S7 Fig A549 Naive and NAFc4 plus Donor 2 NK 5 Fig 4E. (PDF)

S8 Fig A549 Naive and NAFc4 plus Donor 2 NK 2.5 Fig 4F. (PDF)

S9 Fig Acute versus PI Fig 10. (PDF)
