# Supplementary figures and images for: Delivery of a novel membrane-anchored Fc chimera enhances NK cell-mediated killing of tumor cells and persistently virus-infected cells
Source: PLoS One. 2023 May 5;18(5):e0285532. doi: 10.1371/journal.pone.0285532 (PMC10162523; doi:10.1371/journal.pone.0285532)

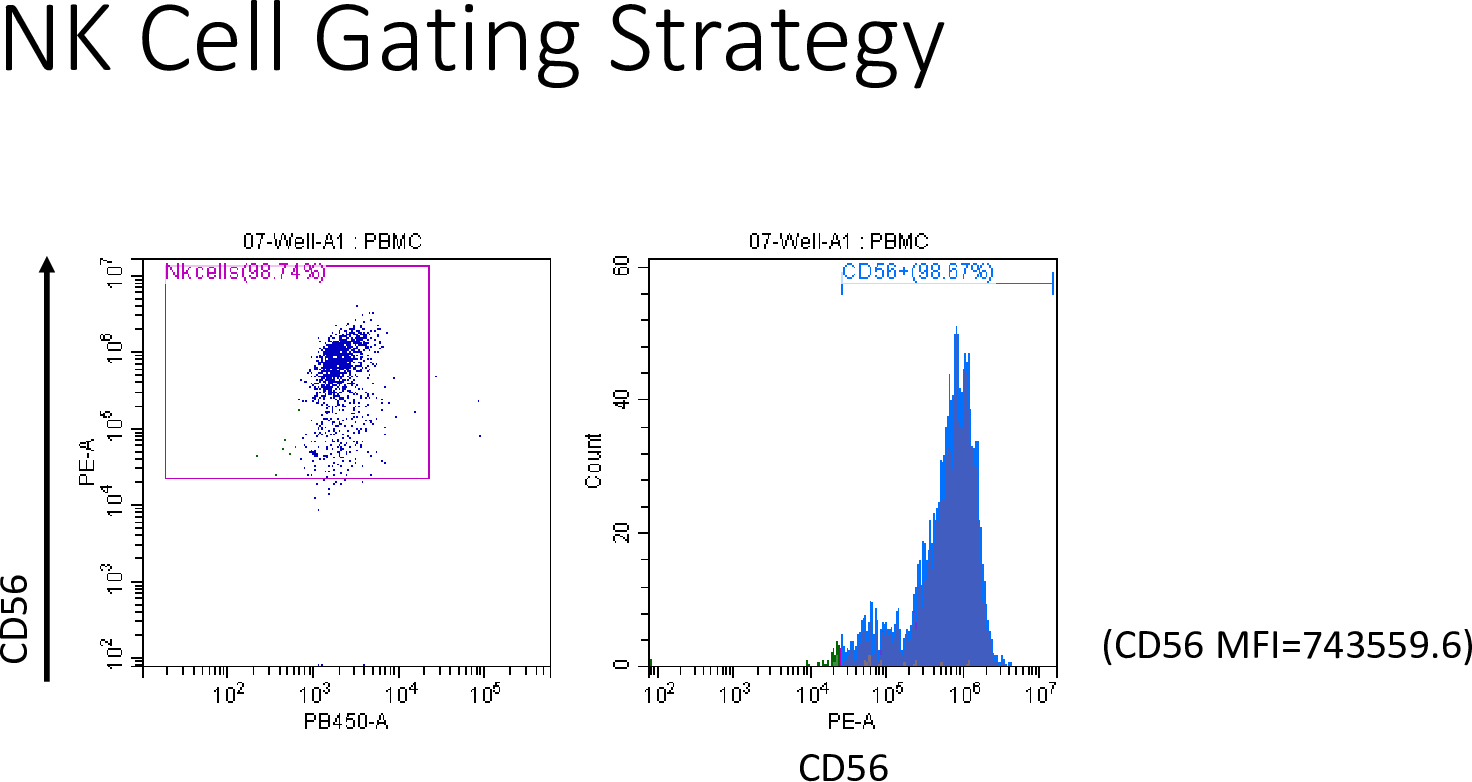

Supplement: S1 Fig — (TIF) [file pone.0285532.s001.tif]

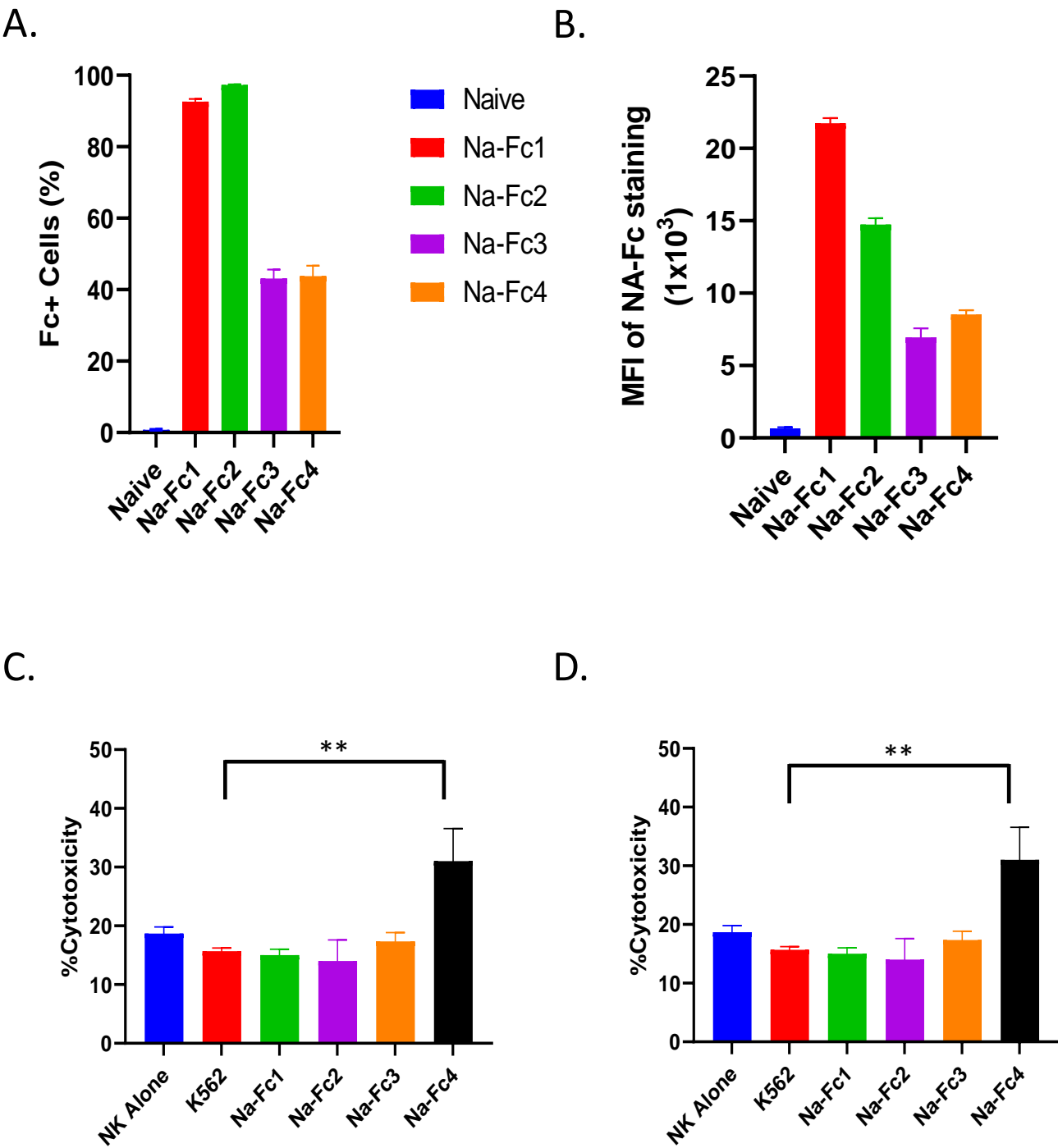

Supplement: S2 Fig — Parental K562 cells and K562 cells expressing one of the four NA-Fc chimeras were stained with stained with antibody to Fc domain. Percent positive (panel A) and MFI (Panel B) for surface Fc expression was assayed by flow cytometry. PM21-NK cells were incubated with cultures of the indicated K562 cells for 20 mins at an E:T of 1.25:1(Panel C) or 45 mins at an E:T of 0.625:1 (Panel D). Percent cytotoxicity was determined using a flow based cytometric assay as described in Material & Methods. Data is from triplicate samples with error bars represent SD. Data was analyzed using two-way ANOVA analysis. * and ** indicate p values of p<0.05 and p<0.01. (PDF) [file pone.0285532.s002.pdf]
