# Supplementary material for: Delivery of a novel membrane-anchored Fc chimera enhances NK cell-mediated killing of tumor cells and persistently virus-infected cells
Source: PLoS One. 2023 May 5;18(5):e0285532. doi: 10.1371/journal.pone.0285532 (PMC10162523; doi:10.1371/journal.pone.0285532)
Supplement: S3 Fig — (PDF) [file pone.0285532.s003.pdf]

	Naïve Target alone			NA-Fc4 Target alone		
0	100	100	100	100	100	100
2	106.3288	104.5852	107.7749	106.1904	106.8411	106.7988
4	112.0275	110.4935	113.7897	110.4588	111.0961	110.5994
6	118.3029	115.9763	118.4835	114.3516	115.022	113.8548
8	123.6236	121.0962	123.3779	118.8398	117.8141	117.0146
10	129.9885	127.9329	128.6902	123.0986	122.303	121.704
12	137.7701	136.1196	137.7459	129.4123	127.6097	127.1926
14	147.5108	144.6615	147.0275	138.2008	136.3461	133.0486
16	156.5823	155.1294	156.2002	145.352	144.4446	140.2588
18	168.1045	165.754	164.6115	156.1411	152.7879	146.9402
20	175.3946	174.2551	172.941	165.8836	158.0488	156.0144
22	183.5404	180.6171	178.5374	174.0468	163.193	160.1142
24	188.707	185.3281	182.6464	181.7005	168.5604	165.1734
26	192.8869	190.0849	188.1218	185.4213	171.0565	166.9601
28	196.3629	193.308	190.4122	190.8113	175.4444	169.6905
30	198.2212	195.0466	192.1059	195.0046	177.0239	173.3401
32	200.513	198.3489	194.4779	198.5791	179.9023	174.8782
34	200.8632	200.4623	196.1399	203.3367	183.0254	177.9724
36	202.6807	199.2996	196.591	207.5007	187.071	182.1094
38	203.6135	201.7333	198.9047	208.4833	189.419	185.0503
