# Supplementary material for: Delivery of a novel membrane-anchored Fc chimera enhances NK cell-mediated killing of tumor cells and persistently virus-infected cells
Source: PLoS One. 2023 May 5;18(5):e0285532. doi: 10.1371/journal.pone.0285532 (PMC10162523; doi:10.1371/journal.pone.0285532)
Supplement: S4 Fig — (PDF) [file pone.0285532.s004.pdf]

	Naïve SKOV3 (E:T- 2.5:1)			NA-Fc4 SKOV3 (E:T- 2.5:1)		
0	100	100	100	100	100	100
2	99.57262	96.49197	96.49197	100.7265	97.93958	97.93958
4	93.25076	91.52261	91.52261	92.52052	88.87843	88.87843
6	89.23364	85.64787	85.64787	83.32126	84.61298	84.61298
8	83.61732	78.3792	78.3792	75.05394	71.8476	71.8476
10	77.93583	72.61908	72.61908	63.29636	61.08246	61.08246
12	73.39104	67.01559	67.01559	54.04954	51.19645	51.19645
14	66.85022	61.55318	61.55318	46.76785	43.15234	43.15234
16	63.48449	60.38367	60.38367	39.12981	36.57225	36.57225
18	59.52941	56.6645	56.6645	31.01015	29.97966	29.97966
20	55.97274	53.79613	53.79613	28.19743	24.14567	24.14567
22	52.68036	49.99251	49.99251	23.77404	19.86993	19.86993
24	49.53763	49.31605	49.31605	20.62949	16.30754	16.30754
26	47.5223	47.7114	47.7114	18.40586	13.97275	13.97275
28	45.55793	45.3259	45.3259	15.77255	11.96037	11.96037
30	43.36153	41.96549	41.96549	11.93558	9.08595	9.08595
32	40.51568	38.12762	38.12762	10.71745	6.909786	6.909786
34	37.17307	35.17767	35.17767	8.384151	5.91654	5.91654
36	33.31126	31.85666	31.85666	8.325623	4.795539	4.795539
38	30.17377	28.00561	28.00561	6.575481	5.014733	5.014733
