# Supplementary material for: Delivery of a novel membrane-anchored Fc chimera enhances NK cell-mediated killing of tumor cells and persistently virus-infected cells
Source: PLoS One. 2023 May 5;18(5):e0285532. doi: 10.1371/journal.pone.0285532 (PMC10162523; doi:10.1371/journal.pone.0285532)
Supplement: S5 Fig — (PDF) [file pone.0285532.s005.pdf]

	Naïve A549 (E:T- 5:1)			NA-Fc4 A549 (E:T- 5:1)		
0	100	100	100	100	100	100
2	98.7396	95.68789	97.83567	95.89972	95.1843	98.74264
4	88.80328	88.49939	96.06225	86.87649	86.15145	91.07821
6	82.88288	84.86596	92.88631	74.59098	75.54867	80.85348
8	80.96859	77.7056	90.58807	62.71668	63.93612	70.04381
10	73.4499	71.31534	85.45223	55.81488	56.19014	60.01413
12	65.51443	64.99523	78.54212	46.29007	44.23033	51.54845
14	63.35834	60.25457	71.29177	38.17789	36.42869	44.33762
16	54.65989	52.22181	63.41837	32.89733	30.75171	37.74509
18	50.09106	49.49927	60.12646	27.54767	26.3999	30.96921
20	46.092	43.84467	54.66404	23.89992	20.91988	27.11151
22	42.74622	41.45854	48.30067	20.16234	17.58025	22.40459
24	38.82019	34.11917	43.83421	16.84893	14.42568	18.58554
26	34.11207	31.23641	40.99664	13.981	11.36584	14.88911
28	30.82532	27.80598	37.81793	12.47014	9.781157	13.73076
30	28.95802	26.03868	34.35336	10.75285	8.516932	12.17853
32	25.09687	25.02239	30.90302	9.076816	7.164008	10.17438
34	23.59208	23.49174	29.5776	7.756503	6.173256	8.577985
36	21.9975	20.90943	26.60157	6.452477	5.003092	7.09441
38	21.76078	18.65599	24.13447	5.295119	4.156972	6.365336
40	19.53087	17.73218	22.80293	4.761686	3.744392	5.699704
42	17.84723	16.8212	20.27052	4.347045	3.258022	4.720413
44	16.71231	14.27695	18.36805	3.672845	2.485029	4.134399
