# Supplementary material for: Delivery of a novel membrane-anchored Fc chimera enhances NK cell-mediated killing of tumor cells and persistently virus-infected cells
Source: PLoS One. 2023 May 5;18(5):e0285532. doi: 10.1371/journal.pone.0285532 (PMC10162523; doi:10.1371/journal.pone.0285532)
Supplement: S6 Fig — (PDF) [file pone.0285532.s006.pdf]

	Naïve A549 (E:T- 2.5:1)			NA-Fc4 A549 (E:T- 2.5:1)		
0	100	100	100	100	100	100
2	101.3838	100.6597	97.90865	99.32898	98.42301	97.34946
4	100.8724	102.2705	93.98684	95.35543	93.4569	94.67944
6	96.25677	97.12742	91.30383	92.51524	86.47446	86.59827
8	92.35682	95.30959	87.36678	85.50256	78.80151	82.48966
10	87.80524	89.3077	84.38847	76.57371	71.26528	74.25103
12	82.79438	88.2569	80.08851	68.82578	62.99514	66.64597
14	73.96294	79.29645	74.43697	59.41753	55.98688	58.22896
16	68.5439	76.69745	68.45731	50.36732	47.53726	49.56131
18	61.70722	67.5557	64.83832	42.37082	41.07625	42.30207
20	58.14845	59.96706	58.54258	34.9774	34.95472	34.73207
22	52.83794	55.62861	55.04058	31.01811	29.80573	30.06122
24	48.3473	49.42848	51.02715	26.59589	25.14737	26.63896
26	44.58116	46.59921	45.95741	23.36798	22.40652	22.8993
28	40.36066	43.21606	41.27618	21.14324	19.58002	19.19855
30	37.71216	39.00777	38.90009	17.53592	17.32596	18.30933
32	33.40174	36.06711	34.88734	15.65941	14.87433	15.22051
34	30.97455	32.29964	33.39084	13.8237	13.23906	13.92432
36	29.13772	30.1955	30.31857	12.61926	11.27174	12.5069
38	26.77073	28.71812	28.00544	10.88062	10.70766	10.7722
40	24.64905	26.15177	24.59836	10.06262	9.596471	10.33825
42	22.5978	24.63817	24.3599	8.969327	7.94091	9.038216
44	20.06738	21.71509	22.14165	7.932386	7.09897	7.766609
