# Supplementary material for: Delivery of a novel membrane-anchored Fc chimera enhances NK cell-mediated killing of tumor cells and persistently virus-infected cells
Source: PLoS One. 2023 May 5;18(5):e0285532. doi: 10.1371/journal.pone.0285532 (PMC10162523; doi:10.1371/journal.pone.0285532)
Supplement: S7 Fig — (PDF) [file pone.0285532.s007.pdf]

	Naïve A549 (E:T- 5:1)			NA-Fc4 A549 (E:T- 5:1)		
0	100	100	100	100	100	100
2	110.4322	109.2378	110.9555	98.00262	101.6881	103.8129
4	112.9084	108.5812	103.674	94.22945	97.30941	98.60376
6	108.6453	99.99618	102.1082	83.28855	87.90572	87.95425
8	100.944	94.83768	95.3986	72.55082	75.29719	77.68408
10	95.96387	84.33788	86.18817	61.30752	69.12029	66.70759
12	88.84229	79.88078	80.70765	49.66478	58.54362	55.89341
14	80.71258	74.41486	72.95185	41.46814	50.7236	46.84982
16	76.03222	65.75417	67.09559	33.8037	40.85777	41.37496
18	68.61228	60.38057	63.47932	29.43843	36.25147	35.82847
20	61.24579	53.69755	55.46669	24.82182	30.84936	31.01261
22	54.54063	50.99031	52.06029	21.12176	27.60812	24.71594
24	50.91671	46.33493	47.06939	18.05457	24.18837	22.3345
26	46.23134	44.13753	42.80777	15.13759	21.9014	20.2612
28	43.58574	42.1861	42.48629	13.4554	19.14098	17.18349
30	40.30015	37.93761	37.0919	12.23813	17.52737	14.67996
32	36.95169	34.96348	34.74949	9.812099	15.94205	12.43508
34	34.82959	31.24264	30.92111	9.822011	14.2712	11.16382
36	31.34274	29.00842	29.21697	8.505838	12.24258	10.50227
38	28.68052	26.92476	27.48608	7.795298	10.7864	9.334501
40	26.78474	24.59949	26.32508	7.768654	8.819747	8.36263
42	24.51467	22.64022	24.5604	7.193345	8.083394	8.105094
44	23.17842	20.77058	23.32242	6.187574	7.649523	6.611796
46	21.24062	19.01709	21.17104	5.736744	6.874723	6.589796
