# Supplementary material for: Delivery of a novel membrane-anchored Fc chimera enhances NK cell-mediated killing of tumor cells and persistently virus-infected cells
Source: PLoS One. 2023 May 5;18(5):e0285532. doi: 10.1371/journal.pone.0285532 (PMC10162523; doi:10.1371/journal.pone.0285532)
Supplement: S8 Fig — (PDF) [file pone.0285532.s008.pdf]

	Naïve A549 (E:T- 1.25:1)			NA-Fc4 A549 (E:T- 1.25:1)		
0	100	100	100	100	100	100
2	113.0171	109.9372	109.9372	113.165	111.3054	108.9058
4	116.5317	113.2199	113.2199	115.5226	112.8579	110.7199
6	116.7235	114.1946	114.1946	113.5817	109.7979	109.325
8	112.8069	113.4094	113.4094	110.648	105.4755	105.5993
10	112.9728	112.8402	112.8402	103.1143	102.3948	101.3077
12	111.652	109.0129	109.0129	96.18919	97.88552	95.09556
14	108.3293	107.3916	107.3916	91.84007	93.03512	91.28205
16	101.5521	102.7305	102.7305	85.32227	87.6792	86.14024
18	99.79529	101.9947	101.9947	78.11179	83.65365	79.80953
20	93.49686	98.99453	98.99453	73.73007	77.93218	73.25607
22	90.63338	94.4558	94.4558	69.52408	73.88293	68.76402
24	84.92986	91.14803	91.14803	61.78049	67.45211	66.11346
26	80.80294	90.18377	90.18377	58.17009	61.77238	60.78369
28	78.79144	86.53623	86.53623	54.44536	59.53446	58.4736
30	78.17872	82.55437	82.55437	49.78675	56.03226	53.97053
32	76.33621	80.20316	80.20316	48.63198	52.70272	53.06955
34	71.10952	78.19773	78.19773	45.54853	49.17169	48.62479
36	67.89012	75.83933	75.83933	43.97471	47.02117	46.61652
38	64.56885	71.4789	71.4789	40.15335	44.27938	44.90748
40	62.20628	67.03735	67.03735	36.26538	42.17014	42.24014
42	59.24579	64.39832	64.39832	33.41539	40.28301	39.41957
44	56.8485	62.1702	62.1702	31.76986	38.25377	38.32792
46	55.70938	60.24617	60.24617	31.29719	35.79078	37.13338
