# Supplementary material for: Delivery of a novel membrane-anchored Fc chimera enhances NK cell-mediated killing of tumor cells and persistently virus-infected cells
Source: PLoS One. 2023 May 5;18(5):e0285532. doi: 10.1371/journal.pone.0285532 (PMC10162523; doi:10.1371/journal.pone.0285532)
Supplement: S9 Fig — (PDF) [file pone.0285532.s009.pdf]

	P/V Acute			P/V PI			LV BFP P/V PI			LV NA-Fc4 P/V PI		
0	100	100	100	100	100	100	100	100	100	100	100	100
2	96.9523	92.96282	97.17402	101.6625	100.543	101.6625	99.04485	98.9081	98.9081	95.06557	93.17059	94.85748
4	88.777	84.98866	85.67857	103.0061	99.15256	103.0061	95.70871	94.94421	94.94421	91.20784	86.98127	87.43066
6	79.44031	76.41576	77.01404	100.743	96.72668	100.743	93.68624	95.19521	95.19521	85.79116	82.29193	82.18887
8	70.37936	68.23629	71.22361	97.97511	94.18349	97.97511	91.65968	92.71887	92.71887	81.39234	77.00482	79.12695
10	65.88554	63.49857	64.17653	96.24432	91.90222	96.24432	90.13689	90.25241	90.25241	77.8148	74.54422	75.99709
12	58.89859	55.39349	58.3898	90.70171	87.82451	90.70171	88.12439	90.33823	90.33823	75.19276	70.10039	70.63358
14	52.37904	49.57526	51.74165	86.44582	83.96733	86.44582	84.7527	86.93127	86.93127	72.25022	66.3836	67.38123
16	44.8333	43.79249	45.30758	82.90355	80.47114	82.90355	82.33539	83.03516	83.03516	68.68781	61.96193	63.12194
18	39.54664	38.34763	41.11819	78.29739	75.62139	78.29739	78.47147	80.36243	80.36243	64.701	57.68093	56.70862
20	35.55571	34.34819	35.44523	74.49313	71.85962	74.49313	69.94112	77.60751	77.60751	58.98138	53.86987	52.26699
22	31.17914	30.56043	31.12241	68.89909	67.40131	68.89909	69.58588	71.57729	71.57729	56.51122	48.64977	47.69955
24	28.10129	27.21771	28.08212	66.68262	65.06681	66.68262	63.92609	67.14382	67.14382	52.12651	44.56448	42.35529
26	25.22683	22.81336	25.4571	61.59104	60.40005	61.59104	60.90709	63.673	63.673	47.07228	42.24583	39.02748
28	22.61615	20.23464	22.4238	58.15916	57.41807	58.15916	56.61744	60.27046	60.27046	44.85906	38.46943	35.09933
30	20.42938	18.04862	20.03727	54.40805	54.40984	54.40805	53.70242	57.39764	57.39764	41.76896	36.11911	31.61538
32	18.23133	16.03436	18.10946	50.43584	50.69733	50.43584	50.25325	53.45153	53.45153	37.96366	33.83911	28.74781
34	16.53253	14.81266	16.12744	47.3065	48.59027	47.3065	46.4561	50.95684	50.95684	35.97866	31.0641	25.89176
36	14.36882	13.18889	14.63059	43.79403	45.42114	43.79403	44.25479	48.18806	48.18806	33.34968	30.21166	24.00604
38	12.89883	12.98513	13.14251	41.40314	41.87172	41.40314	41.58956	46.01218	46.01218	31.84213	28.57837	22.64402
40	11.8998	11.16286	12.56297	38.36805	39.31563	38.36805	39.03522	43.43483	43.43483	29.40684	27.39659	22.26022

	P/V Acute			P/V PI			LV BFP P/V PI			LV NA-Fc4 P/V PI		
0	100	100	100	100	100	100	100	100	100	100	100	100
2	97.90228	93.99267	97.58401	100.9992	100.9992	101.5065	101.2765	100.9992	100.9992	100.3612	98.45078	100.4663
4	92.61987	90.73821	93.94681	100.2821	100.2821	99.80119	102.4623	100.2821	100.2821	100.7364	99.38173	98.23968
6	87.84743	83.83276	86.97322	101.9126	101.9126	101.6728	102.1767	101.9126	101.9126	97.88046	96.56293	95.77228
8	83.08293	78.75562	83.23396	102.28	102.28	101.0304	101.2896	102.28	102.28	95.59077	91.43916	92.81224
10	75.50536	70.17567	77.55138	98.78899	98.78899	102.1266	99.12473	98.78899	98.78899	91.03018	88.34248	89.34289
12	69.56721	63.94569	70.286	97.12822	97.12822	99.6622	95.52542	97.12822	97.12822	88.35911	84.54449	83.56039
14	64.49741	60.00188	64.72525	93.05682	93.05682	97.86292	92.49553	93.05682	93.05682	82.6351	80.99934	78.3917
16	60.36074	53.66639	58.61351	90.5593	90.5593	95.79426	87.17706	90.5593	90.5593	79.94133	74.93778	75.45659
18	54.36266	49.48619	54.76886	85.17412	85.17412	92.17509	81.9665	85.17412	85.17412	73.50761	71.48681	69.91739
20	50.63577	44.45847	48.94645	81.008	81.008	88.60022	79.2001	81.008	81.008	69.13444	68.32714	66.19572
22	44.72052	40.08313	45.89552	76.90869	76.90869	87.02378	73.28113	76.90869	76.90869	63.62849	62.49019	63.63963
24	40.88686	35.26937	41.81266	74.22115	74.22115	86.10017	70.08302	74.22115	74.22115	59.00634	57.882	58.80478
26	37.3656	33.5952	38.53778	73.55504	73.55504	85.32569	66.45918	73.55504	73.55504	53.86576	55.89992	57.32205
28	34.22134	31.03172	35.67683	73.34438	73.34438	85.33022	65.05411	73.34438	73.34438	50.07214	53.4049	54.00634
30	31.33193	28.6661	33.60699	71.61434	71.61434	83.8218	63.78647	71.61434	71.61434	46.29263	52.07263	51.61137
32	28.78359	25.97414	30.6896	72.44389	72.44389	84.02736	61.49155	72.44389	72.44389	45.09402	48.88985	48.98413
34	26.70941	24.29214	27.58562	71.03221	71.03221	84.85644	60.30804	71.03221	71.03221	41.50968	47.40945	47.71778
36	23.86262	22.67205	25.74181	69.60207	69.60207	84.06636	56.92765	69.60207	69.60207	38.63119	44.08802	45.85182
